# Multi-Omics Analyses Identify Signatures in Patients with Liver Cirrhosis and Hepatocellular Carcinoma

**DOI:** 10.3390/cancers15010210

**Published:** 2022-12-29

**Authors:** Ming-Wei Lai, Yu-De Chu, Chao-Wei Hsu, Yi-Cheng Chen, Kung-Hao Liang, Chau-Ting Yeh

**Affiliations:** 1 Department of Pediatrics, Chang Gung Memorial Hospital, Linkou Branch and Chang Gung University College of Medicine, Taoyuan 333, Taiwan; 2 Liver Research Center, Chang Gung Memorial Hospital, Linkou Branch and Chang Gung University College of Medicine, Taoyuan 333, Taiwan; 3 Department of Hepatogastroenterology, Chang Gung Memorial Hospital, Linkou Branch and Chang Gung University College of Medicine, Taoyuan 333, Taiwan; 4 Department of Medical Research, Taipei Veterans General Hospital, Taipei 112, Taiwan; 5 Molecular Medicine Research Center, College of Medicine, Chang Gung University, Taoyuan 333, Taiwan

**Keywords:** microbiota, virome, metabolomics, cytokine/chemokine, liver cirrhosis, hepatocellular carcinoma

## Abstract

**Simple Summary:**

Gut dysbiosis with an impaired intestinal barrier is differentially associated with liver diseases and, therefore, alters the systemic immunological milieu through the gut–liver axis. As a result, it plays a prominent role in liver inflammation, fibrosis, and carcinogenesis. Here, a list of network features common to patients with liver cirrhosis or hepatocellular carcinoma (HCC), regardless of etiology, was identified. In addition, disease-specific signatures were identified. This study provides novel insights that suggest alterations in gut microbial/viral composition may either confer pre-HCC disorders or contribute to the metabolic reprogramming and/or inflammatory microenvironment for HCC development.

**Abstract:**

Gut bacterial/viral dysbiosis, changes in circulating metabolites, and plasma cytokines/chemokines have been previously associated with various liver diseases. Here, we analyzed the associations between fecal microbial composition, circulating metabolites, and plasma cytokines/chemokines in patients with liver cirrhosis (LC) and hepatocellular carcinoma (HCC). We recruited 10 HCC patients, 18 LC patients, and 17 healthy individuals. Their stool samples were used for gene sequencing of bacterial 16S rRNA and viral genomes, while plasma samples were utilized for the determination of endotoxin, zonulin, metabolite, and cytokine/chemokine levels. Dysbiosis was observed among gut bacteria and viruses, with significant changes in abundance at the genus and species levels, respectively. However, no differences were found between cohorts in the alpha and beta diversity. Plasma lipopolysaccharides and zonulin, but not trimethylamine N-oxide, were progressively increased in LC and HCC subjects. Profiling plasma metabolites and selected cytokines/chemokines revealed differential changes in the LC and HCC cohorts. Following joint correlation and correlation network analyses, regardless of etiology, common network signatures shared by LC and HCC patients were characterized by the gut virus Stenotrophomonas virus DLP5 and the uncultured Caudovirales phage, plasma metabolites pyruvic acid and acetic acid, and plasma cytokines/chemokines eotaxin and PDGF-AB/BB, respectively. Additionally, LC- and HCC-specific correlation networks were also identified. This study provides novel insights into altered gut microbial/viral composition that may contribute to pre-HCC disorders, metabolic reprogramming, or inflammatory microenvironments for hepatocarcinogenesis.

## 1. Introduction

Hepatocellular carcinoma (HCC) accounts for more than 80% of patients and is the most common type of liver cancer, the 7th most common malignancy, and the 2nd leading cause of cancer-related mortality worldwide [1]. Chronic liver inflammation and liver cirrhosis (LC) due to alcohol abuse, hepatitis B virus (HBV) or hepatitis C virus (HCV) infection, and nonalcoholic fatty liver disease (NAFLD)/nonalcoholic steatohepatitis (NASH) are the main known etiological factors in the development of HCC [2,3]. Since the liver and gut are anatomically and physiologically linked through the biliary-enteral connection and the portal venous system, it is often referred to as the gut–liver axis. An inappropriate diet (e.g., high fat and alcohol), xenobiotics, or impaired bile secretion can disrupt this axis. In this case, the bidirectional crosstalk is disturbed, leading to an imbalance of the intestinal microbiota, disruption of the intestinal barrier, and translocation of microbes and their derivatives to the liver via portal vein. In the context of the dysregulation of the gut–liver axis, the hepatic microenvironment can transform into a pro-inflammatory, senescent, and tumor-promotion milieu, superimposed on existing risk factors for hepatocarcinogenesis [4]. Therefore, in addition to these canonical risk factors, the pathogenesis of various liver diseases (e.g., NAFLD and NASH) and the carcinogenesis of HCC have recently been linked to the dysregulation of the gut–liver axis due to gut dysbiosis and increased intestinal permeability in abnormal lifestyle conditions, such as a long-term high-fat diet [4].

The intestinal barrier is well known for its role in preventing the escape of microorganisms and molecules from the gut lumen. It is composed of enterocytes that are tightly bound to neighboring cells by apical junction proteins, such as claudins, occludins, E-cadherins, desmosomes, and adhesion molecules. The release of components caused by gut inflammation and dysbiosis disrupts the integrity of the intestinal barrier and leads to increased gut permeability. Therefore, with increased gut permeability, intestinal microorganisms and their derivatives can migrate to the liver and contribute to the worsening of liver diseases (for a review see reference [4]). In recent years, increased intestinal permeability has been considered a hallmark of LC, thereby exposing the liver to many bacteria and microbial components of a leaky gut, such as lipopolysaccharide (LPS) [4]. The interaction between LPS and hepatocyte Toll-like receptor-4 (TLR4) has been shown to be crucial in hepatocarcinogenesis through inflammation, chronic liver injury, and liver fibrosis [5,6]. In addition, emerging evidence supports the idea that distinct gut microbiome and virome composition signatures can identify patients with liver disease and show its severity, including LC and HCC [7,8,9,10,11]. Additionally, studies integrating the intestinal microbiota and metabolites or cytokines/chemokines have also shown that their dysregulation plays a determining role in the pathogenesis of liver diseases [8,9,12]. Furthermore, disturbances in their content are associated with intrahepatic and peripheral immune responses [8,9,12].

In patients with HBV-related LC, the dysbiosis of *Bacteroidetes*, *Firmicutes*, *Enterobacteriaceae,* and *Lachnospiraceae* has previously been observed in the gut microbiota [13]. Furthermore, the *Bacteroides/Enterobacteriaceae* ratio was also significantly reduced progressively in asymptomatic carriers, and chronic hepatitis connected to decompensated LC was related to HBV infection [14]. On the other hand, in patients with NAFLD-related HCC, the increased *Bacteroides* and *Ruminococcaceae* and the decreased *Bifidobacterium* were, respectively, correlated with intestinal inflammatory markers and increased circulating proinflammatory cytokines, such as interleukin-8 (IL8), IL13, chemokine (C-C motif) ligand (CCL)-3 (or MIP-1a), CCL4 (or MIP-1b), and CCL5 (or RANTES) [8]. Regarding the interplay between intestinal bacterial metagenome and virome, bacteriophage abundance has also been shown to be inversely correlated with the abundance of autochthonous bacteria in LC patients [11,15]. However, the relationship between intestinal bacterial and viral abundance in HCC patients remains unclear.

Although an increasing number of studies have shown the associations between any two or three of the microbial metagenome, virome, metabolites, and cytokines/chemokines [7,8,9,10,11,12,13,14,15,16], their concurrent association in a single study has never been studied before. Except for one study that combined multiple omics tools to investigate associations between bacterial metagenomes, cytokines/chemokines, peripheral blood mononuclear cells (PBMCs), and metabolites in patients with NAFLD-related HCC [16], no observational studies have investigated associations between bacterial metagenome, virome, cytokine/chemokine, and metabolite profiles in patients with LC and HCC. Additionally, most studies have focused on a single etiology in patients, particularly for NAFLD/NASH-related LC or HCC [7,8,9,11,16]. In this study, we sought to explore common or exclusive signatures of microbial metagenome, virome, metabolite, and cytokine/chemokine in LC and HCC patients with multiple etiologies compared to healthy controls.

## 2. Materials and Methods

### 2.1. Patients

A total of three cohorts were prospectively enrolled in this study. The first included 18 patients with LC; the second included 10 patients with LC who developed early-stage HCC; the third included 17 healthy individuals as controls, with a body mass index (BMI) between 18.5 and 27.0, alanine aminotransferase (ALT) ≤ 36 U/L, age ≥ 40 years, and general health without systemic disease. Diagnosis of LC was based on clinical portal hypertension and supporting images, including ultrasonography (Fibroscan), abdominal computed tomography (CT), and magnetic resonance imaging (MRI), or definitive liver pathology. HCC was confirmed by imaging (CT scan, MRI, or angiography) and histological or cytological studies. Fecal and blood samples were obtained from all recruited subjects, and their clinical parameters were recorded at the time of recruitment. For all participants, no exposure to antibiotics or probiotics was allowed for at least three months before stool collection. This study was approved by the Institutional Review Board of Chang Gung Memorial Hospital, Linkou branch (Approval number: 201800985B0, 23 July 2018). All participants signed an informed consent form before entering this study.

### 2.2. Fecal Bacterial 16S rRNA Gene Sequencing

DNA was extracted from 200 mg of feces using the QIAamp DNA Stool Mini Kit (Qiagen, Hilden, Germany). Bacterial variable regions V3-V4 of the 16S rRNA gene were amplified using primers 16S_F: 5′-TCGTCGGCAGCGTCAGATGTGTATAAGAGACAGCCTACGGGNGGCWGCAG-3′ and 16S_R: 5′-GTCTCGTGGGCTCGGAGATGTGTATAAGAGACAGGACTACHVGGGTATCTAATCC)-3′, according to the MiSeq rRNA Amplicon Sequencing protocol (Illumina, San Diego, CA). After purification, amplicon pools were prepared for sequencing using the Truseq DNA library preparation kit (Illumina). Approximately 100 ng of purified amplicon pools were repaired to generate blunt-ended, 5′-phosphorylated DNA and performed an A-tailing reaction compatible with the adapter ligation strategy. The ligation products were purified by AMPure XP beads, quantified, and diluted to an equimolar concentration of 4 nM. Multiple samples were pooled before sequencing on the Illumina MiSeq platform to generate paired-end reads with 300 bases in length.

The 16S rRNA gene amplicon data were analyzed using QIIME 2 software and the DADA2 pipeline. Raw sequences were demultiplexed, quality filtered, denoised, and pair-end merged, and chimera was removed using pipelines. The reads were then clustered into amplicon sequence variants (ASVs) with 97% identity. The DADA2 classifier was applied to compare ASVs to the training set of classified sequences, including GreenGenes, SILVA, NCBI, eHOMD, and UNITE databases [17,18,19,20].

### 2.3. Fecal DNA Virus Genome Sequencing

Details of virus-like particles purification and DNA virus genome isolation are available in the Supplementary Materials and Methods. After isolation of DNA virus genome, whole genome amplification was performed using Illustra™ GenomiPhi™ V2 kit (GE Healthcare) reagents and protocols to generate sufficient material for library construction. The amplified products were then pooled and cleaned using Dneasy kit (QIAGEN). A total of 1 μg of cleaned DNA was used for library construction using Illumina Nextera DNA Flex Library Prep (Illumina), according to the manufacturer’s instructions. The library was sequenced on the Illumina NovaSeq 6000 platform with paired reads of 150 bp in length.

The obtained raw data were transformed into raw sequenced reads using CASAVA base calling and stored in FASTQ format. The resulting raw paired-end reads were filtered using Trimmomatic to remove low-quality reads, trim adaptor sequences, and eliminate poor-quality bases with the following parameters: LEADING:3, TRAILING:3, SLIDINGWINDOW:4:20, MINLEN:100, and AVGQUAL:20 [21]. Cleaned reads were used to filter contaminating host sequences using Bowtie2 [22], assemble contigs using MEGAHIT [23], predict open reading frames using Prodigal [24], construct nonredundant gene catalog using CD-HIT [25], map to initial gene catalog using BWA [26], and create BAM files using SAMtools [27]. BAM files were used to calculate the coverage of each sample using the MetaBAT2 pipeline [28]. Genome percentage completeness and contamination of all bins were assessed using CheckM [29]. The obtained unigenes were blasted against the NCBI Refseq database using DIAMOND [30,31]. Taxonomic assignments were determined using the lowest common ancestor (LCA) algorithm. Gene annotations were conducted by aligning sequences across multiple databases using DIAMOND, HMMER, and other annotators specified in the database.

### 2.4. Plasma Endotoxemia Evaluation and Zonulin Measurement

Plasma samples were collected for LPS measurement using the PYROGENT™-5000 and Kinetic-QCL™ assays (Lonza Walkersville, Inc. Walkersville, MD, USA). The endotoxin concentrations in the plasma samples were determined from a standard curve. Plasma zonulin levels were measured using the IDK Zonulin ELISA Kit (Immundiagnostik AG, Salem, NH, USA), according to the manufacturer’s instructions. The assay was based on the competitive ELISA method. A biotinylated zonulin tracer was used as a competitor. Free zonulin in the sample competes with the tracer for binding to anti-zonulin antibodies immobilized on the microtiter plate wells. The standards and controls were assayed simultaneously with the samples.

### 2.5. Plasma Aqueous Phase Metabolite Measurement

Plasma samples were prepared for metabolomic analysis based on nuclear magnetic resonance (NMR) by mixing 3:1 (*v*/*v*) of samples and citrate buffer (pH 4.4) using an automated mixer on board. The analysis was performed on an automated high-throughput NMR platform with an Agilent spectrometer 400 MHz (9.4 T), a 4 mm indirect detection probe, and a fixed flow cell (Agilent Technologies, Santa Clara, CA, USA). Data were acquired and processed using Topspin 3.6 (Bruker Biospin), and experiments were performed under automation by the IconNMR program (Bruker Biospin) using the standard pulse sequence, cpmgpr1d (Bruker Biospin).

### 2.6. Plasma Cytokine/Chemokine Profiles

Plasma samples were collected, diluted 1:4, and performed in duplicate for systemic cytokine/chemokine responses. A panel of 27 cytokine/chemokine and growth factors, including IL-1b, IL-1 receptor antagonist (IL-1ra), IL-2, IL-4, IL-5, IL-6, IL-7, IL-8, IL-9, IL-10, IL-12, IL-15, IL-17, fibroblast growth factor (FGF) beta, granulocyte colony-stimulating factor (G-CSF), granulocyte-macrophage colony-stimulating factor (GM-CSF), interferon gamma (IFN-γ), MCP-1 (CCL2), MIP-1a (CCL3), MIP-1b (CCL4), RANTES (CCL5), Eotaxin (CCL11), IP-10 (C-X-C motif chemokine ligand (CXCL) 10), platelet-derived growth factor (PDGF) AB/BB, vascular endothelial growth factor-a (VEGF-a), and tumor necrosis factor (TNF) alpha, were measured using the MILLIPLEX MAP Human Cytokine/Chemokine Magnetic Bead Panel (MerkMillipore, Darmstadt, Germany), a multiplex, magnetic bead-based immunoassay.

### 2.7. Statistical Analysis

Parametric data were expressed as mean ± standard deviation and compared using student’s *t*-test if data were normally distributed, whereas if data were not normally distributed, it was presented as median (range) and compared using the Mann–Whitney test. Dichotomized data were presented as numbers and percentages (%) and compared using the chi-square test. The omics data of each cohort were compared using the Kruskal–Wallis test for three groups. Then, Dunn’s post hoc analysis for multiple comparisons in any two groups was performed to identify the specific group differences with adjusted *p* values. In all comparisons, a *p* (and adjusted *p*) value <0.05 was considered statistically significant. Alpha diversity was assessed using the ACE, Chao 1, Shannon, and Simpson indices. Beta diversity was assessed using weighted (Bray–Curtis) and unweighted (UniFrac) distance matrices. Correlation analysis was performed using the Pearson correlation. Heatmap and correlation coefficient plot were plotted using the SRplot, an online platform for data analysis and visualization (https://www.bioinformatics.com.cn/srplot) (accessed on 20 October 2022).

## 3. Results

### 3.1. Comparison of Study Cohort Characteristics

The aim of this study was to explore common or exclusive signatures of microbial metagenome, virome, metabolite, and cytokine/chemokine in LC and HCC patients with different etiologies compared to healthy controls. A total of three cohorts were enrolled prospectively, including healthy controls, LC, and HCC. A total of 17 healthy subjects were included as controls, with BMI between 18.5 and 27.0, ALT level ≤36 U/L, age ≥40 years, and general health without systemic disease. A total of 18 patients with stable LC were included in the LC cohort. A total of 10 patients with LC who developed early-stage HCC were included in the HCC cohort. Patients enrolled in the HCC cohort were recruited before any anti-cancer therapy. The clinical parameters of each cohort at enrollment are presented in Table 1. Younger ages were noted in the controls (*p* = 0.024 and <0.001 when compared to the LC and HCC cohorts, respectively), while there was no difference when comparing the age between the LC and HCC cohorts. The lowest BMI was also found in the control group, although a significant difference was only observed between the control and LC cohorts (*p* = 0.002). The main etiologies in the LC and HCC cohorts were chronic HBV infection (61.1% in the LC and 60% in the HCC cohort) and chronic HCV infection (36.4% in the LC and 40% in the HCC cohort). No significant difference was found between the etiology in the LC and HCC groups. Higher levels of AST (*p* = 0.007) and ALT (*p* = 0.013) were observed only in patients with HCC compared to controls, possibly due to disease severity. In patients in the LC and HCC groups, only the lower albumin level in the HCC group differed significantly (*p* < 0.001) but not the other characteristics. Notably, although some patients in the LC and HCC groups were taking oral antiviral drugs against HBV or HCV, the distribution of patients receiving antiviral drugs was not significantly different between the two groups. Furthermore, the Child–Pugh score, which indicates the severity of cirrhosis, was not significantly different between the two groups, although the score increased slightly in the HCC group.

### 3.2. 16S rRNA Gene Composition of Gut Bacteria in Subjects

**Figure 1:**
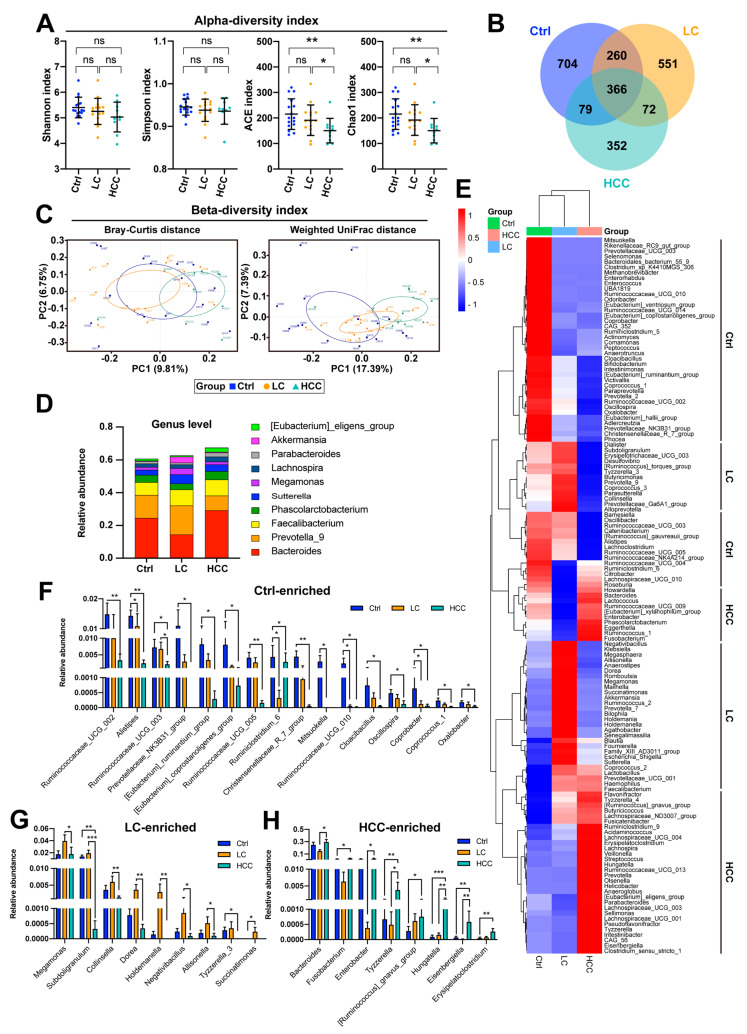
Dysbiosis of gut bacteria in patients with LC and HCC. (**A**) Alpha diversity calculated using the Shannon, Simpson, ACE, and Chao1 indices are shown. The comparison was made using the Kruskal–Wallis test for three groups, and the post hoc Dunn’s test for two groups. *, *p* < 0.05; **, *p* < 0.01; ns, no significance. (**B**) Venn diagram shows the number of bacterial OTUs shared between the three cohorts. (**C**) Beta diversity calculated using the Bray–Curtis method and the weighted UniFrac method are shown. (**D**) The mean abundances of the ten most abundant gut bacteria genera in the three cohorts are shown. (**E**) Mean abundances of bacteria genera in the three cohorts are analyzed using heatmap. Comparison of abundances between cohorts for candidates enriched in the (**F**) control cohort, (**G**) the LC cohort, and (**H**) the HCC cohort. Comparisons were made using the Kruskal–Wallis test for three groups and the Dunn’s test for two groups. *, *p* < 0.05; **, *p* < 0.01; ***, *p* < 0.001.

In the control, LC, and HCC cohorts, an average of 99,098, 97,427, and 92,930 raw pair-end reads, as well as an average of 71,603, 71,601, and 68,446 cleaned reads, were obtained, respectively. Alpha diversity analysis showed that fecal bacterial richness was significantly lower in the HCC cohort when the ACE and Chao1 indices were used (Figure 1A). However, using the Shannon and Simpson indices, no differences were found in terms of compound richness and evenness between cohorts.

A Venn diagram showing the overlap between groups demonstrated that the three groups shared 366 out of 2384 operational taxonomic units (OTUs), 626 of 2032 OTUs were shared between the control and LC cohorts, 445 of 1833 OTUs were shared between controls and the HCC cohort, and 438 out of 1680 OTUs were shared between the LC and HCC cohorts (Figure 1B). Notably, 704, 551, and 352 OTUs in the control, LC, and HCC cohorts were unique, respectively.

To understand the differences in fecal bacterial composition between samples, beta diversity was assessed using principal coordinates analysis (PCoA). As shown in Figure 1C, sample spacing was not statistically significant among the three cohorts using either the Bray–Curtis method or the weighted UniFrac method.

Although the alpha and beta diversity of gut bacteria were not significantly different between cohorts, the mean abundances of the ten predominant genera among these three cohorts were clearly different (Figure 1D). To understand which genera were enriched in a particular cohort, the average abundance of bacterial genera with an average relative abundance greater than 0.01% across the three cohorts was analyzed using the heatmap (Figure 1E). All these bacterial genera were divided into three groups according to which genera were enriched in the control, LC, and HCC cohorts, respectively. Further examination of the statistical significance between two of these three cohorts revealed that *Ruminococcaceae UCG 002, Alistipes, Ruminococcaceae UCG 003, Prevotellaceae NK3B31 group, Eubacterium ruminantium group, Eubacterium coprostanoligenes group, Ruminococcaceae UCG 005, Ruminiclostridium 6, Christensenellaceae R 7 group, Mitsuokella, Ruminococcaceae UCG 010, Cloacibacillus, Oscillospira, Coprobacter, Coprococcus 1,* and *Oxalobacter* were significantly enriched in the control (Figure 1F and Table S1); *Megamonas, Subdoligranulum, Collinsella, Dorea, Holdemanella, Negativibacillus, Allisonella, Tyzzerella 3,* and *Succinatimonas* were significantly enriched in the LC cohort (Figure 1G and Table S1); and *Bacteroides, Fusobacterium, Enterobacter, Tyzzerella, Ruminococcus gnavus group, Hungatella, Eisenbergiella,* and *Erysipelatoclostridium* were significantly enriched in the HCC group (Figure 1H and Table S1).

Linear discriminant analysis (LDA) Effect Size (LEfSe), an algorithm for high-dimensional biomarker discovery, was also used to identify potential bacterial biomarkers in the LC and HCC cohorts. Cladogram shows differentially enriched taxonomic clades (Figures S1 and S2). Among them, the bacterial genera *Coprobacter, Alistipes, Christensenellaceae R 7 group, Ruminiclostridium 6, Ruminococcaceae UCG 010*, and *Oxalobacter*; *Dorea, Subdoligranulum, Holdemanella*, and *Megamonas*; and *Eisenbergiella, Hungatella, Tyzzerella, Dielma, Erysipelatoclostridium*, and *Selenomonas 3* were enriched in the control, LC, and HCC cohorts, respectively. The LDA score histogram showed potential biomarkers (scores ≥ 3) of *Alistipes, Christensenellaceae R 7 group, and Ruminiclostridium 6* in the control cohort; *Megamonas, Subdoligranulum, Dorea, and Holdemanella* in the LC cohort; and *Bacteroides fragilis* in the HCC cohort.

### 3.3. Comparison of Gut Viral Community in Subjects

**Figure 2:**
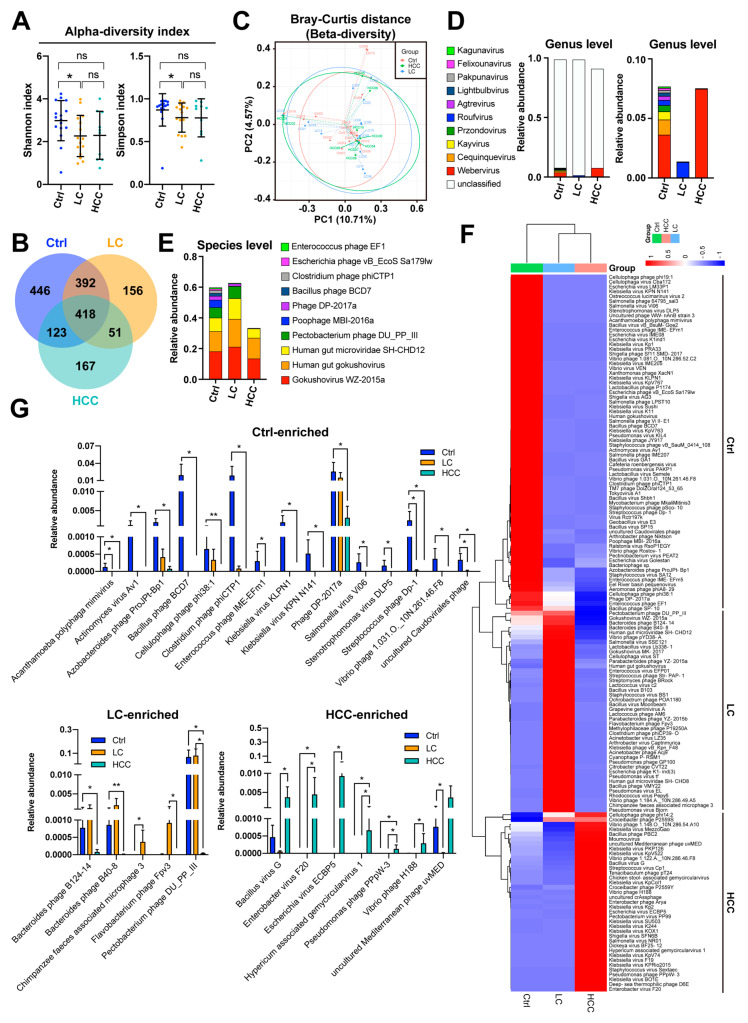
Dysbiosis of gut viruses in patients with LC and HCC. (**A**) Alpha diversity calculated using the Shannon and Simpson indices are shown. Comparison was made using the Kruskal–Wallis test for three groups, and the post hoc Dunn’s test for two groups. *, *p* < 0.05; ns, no significance. (**B**) Venn diagram shows the number of viral OTUs shared between the three cohorts. (**C**) Beta diversity calculated using the Bray–Curtis method is shown. Mean abundances of the ten most abundant gut viruses at (**D**) genus level and (**E**) species level in the three cohorts are shown. (**F**) Mean abundances of gut viral species in the three cohorts are analyzed using heatmap. (**G**) Comparison of abundances between cohorts for candidates enriched in the control cohort (upper panel), the LC cohort (lower left panel), and the HCC cohort (lower right panel). Comparisons were made using the Kruskal–Wallis test for three groups and the post hoc Dunn’s test for two groups. *, *p* < 0.05; **, *p* < 0.01.

On average 66,729, 73,705, and 139,508 of cleaned reads were, respectively, obtained in the control, LC, and HCC cohorts, respectively. The alpha diversity analysis showed that the compound richness and evenness of the cohorts using the Shannon and Simpson indices were significantly different between the control and LC cohorts, but not in the rest (Figure 2A). The Venn diagram showing the overlap between groups demonstrates that 418 of 1753 OTUs were shared by the three groups, 810 of 1586 OTUs were shared between the control and LC cohorts, 541 of 1597 OTUs were shared between the control and HCC cohorts, and 469 of 1307 OTUs were shared between the LC and HCC cohorts (Figure 2B). Notably, 446, 156, and 167 OTUs were unique to the control, LC, and HCC cohorts, respectively. To understand the differences in fecal viral composition between samples, beta diversity was estimated using PCoA. As shown in Figure 2C, the distance between samples was not statistically significant in the three cohorts using the Bray–Curtis method.

Although the diversity of the gut virome did not differ significantly between cohorts, the mean abundance of the top 10 dominant genera and species varied significantly between the three cohorts (Figure 2D,E). To understand which species were enriched in specific groups, the mean virus species abundance, with relative abundance greater than 0.01% across the three cohorts, was assessed using heatmap analysis (Figure 2F). All these gut virus species were divided into three groups, enriched in the control, LC, and HCC cohorts, respectively. Further examination of the statistical significance between two of these three cohorts revealed that Acanthamoeba polyphaga mimivirus, Actinomyces virus Av1, Azobacteroides phage ProJPt-Bp1, Bacillus phage BCD7, Cellulophaga phage phi38:1, Clostridium phage phiCTP1, Enterococcus phage IME-EFm1, Klebsiella virus KLPN1, Klebsiella virus KPN N141, Phage DP-2017a, Salmonella virus Vi06, Stenotrophomonas virus DLP5, Streptococcus phage Dp-1, Vibrio phage 1.031.O._10N.261.46.F8, and uncultured Caudovirales phage were significantly enriched in the control cohort; Bacteroides phage B124-14, Bacteroides phage B40-8, Chimpanzee-feces-associated microphage 3, Flavobacterium phage Fpv3, and Pectobacterium phage DU_PP_III were significantly enriched in the LC cohort; and Bacillus virus G, Enterobacter virus F20, Escherichia virus ECBP5, Hypericum-associated gemycircularvirus 1, Pseudomonas phage PPpW-3, Vibrio phage H188, and uncultured Mediterranean phage uvMED were significantly enriched in the HCC cohort (Figure 2G and Table S2). Although no differentially abundant viral genera were identified for the enrolled cohorts shown in the cladogram (Figure S3), the LDA scores showed potential biomarkers (score ≥3) of Streptococcus phage Dp-1 in the control; Kochikohdavirus in the LC; and the Enterobacter virus F20 in the HCC cohort, respectively (Figure S4).

### 3.4. Increased Levels of LPS and Zonulin, but Not Trimethylamine N-oxide (TMAO) in the LC and HCC Subjects

**Figure 3:**
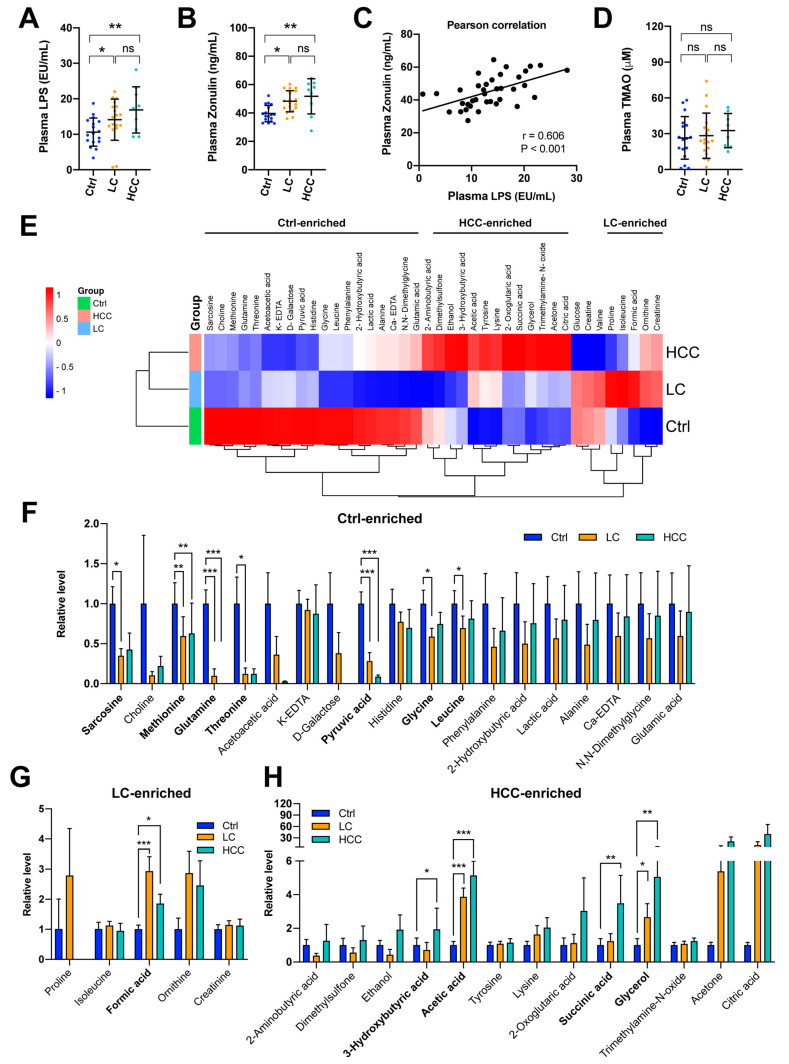
Gut permeability is associated with endotoxemia and plasma metabolite reprogramming in patients with LC and HCC. Comparison of plasma (**A**) lipopolysaccharide (LPS) and (**B**) Zonulin between cohorts. *p* values were obtained using the Kruskal–Wallis test for three groups, and the post hoc Dunn’s test for two groups. *, *p* < 0.05; **, *p* < 0.01; ns, no significance. (**C**) The correlation of endotoxemia (LPS) and gut permeability (Zonulin) by the Pearson’s test. (**D**) Comparison of plasma trimethylamine-N-oxide (TMAO) levels between cohorts. *p* values were obtained using the Kruskal–Wallis test for three groups and the post hoc Dunn’s test for two groups. ns, no significance. (**E**) Mean levels of plasma metabolites in the three cohorts are analyzed using heatmap. Comparison of plasma metabolite levels between cohorts for candidates enriched in (**F**) the control cohort, (**G**) the LC cohort, and (**H**) the HCC cohort. The comparisons were made using the Kruskal–Wallis test for three groups and the post hoc Dunn’s test for two groups. *, *p* < 0.05; **, *p* < 0.01; ***, *p* < 0.001.

Defects in the intestinal epithelium are thought to disrupt gut barrier function, which may lead to increased intestinal permeability and possibly leaky gut, resulting in bacterial products, such as LPS, entering the bloodstream, thus causing enterohepatic circulation via the portal vein and increasing plasma LPS levels (termed endotoxemia) [32]. Therefore, to understand whether the plasma levels of LPS and a biomarker of intestinal permeability, zonulin [33], differed between groups, their levels were evaluated and compared. As shown in Figure 3A,B, the plasma concentrations of LPS (control: 10.6 ± 4.0 (EU/mL); LC: 14.1 ± 5.8 (EU/mL); HCC: 16.9 ± 6.5 (EU/mL)) and zonulin (control: 39.6 ± 5.7 (ng/mL); LC: 48.3 ± 7.4 (ng/mL); HCC: 51.8 ± 12.4 (ng/mL)) gradually increased in the LC and HCC cohorts. Furthermore, their levels were positively correlated (Figure 3C).

Plasma concentrations of a lipid-derived metabolite TMAO are considered elevated in individuals with dysbiosis of the gut, which may be an emerging causative factor of several diseases, including liver disease [34]. Therefore, the level of TMAO was assessed. However, as shown in Figure 3D, unfortunately, plasma TMAO levels were not statistically different between the cohorts.

### 3.5. Signature of Metabolic Changes in Subjects

To understand whether plasma metabolite changes could be markers for LC and HCC, aqueous phase-derived metabolite changes were examined. As shown in Figure 3E, the average levels of metabolites in the aqueous phase were summarized. All of these plasma metabolites were classified into three groups, which were enriched in the control, LC, and HCC cohorts, respectively. Further examination of the statistical significance between two of these three groups revealed that sarcosine, methionine, glutamine, threonine, pyruvic acid, glycine, and leucine were significantly enriched in the control cohort (Figure 3F); formic acid was enriched in the LC group (Figure 3G); and 3-hydroxybutyric acid, acetic acid, succinic acid, and glycerol were significantly enriched in the HCC cohort (Figure 3H). The details of the metabolite comparison are listed in Table 2.

### 3.6. Profiling of a Panel of Cytokines/Chemokines in Subjects

**Figure 4:**
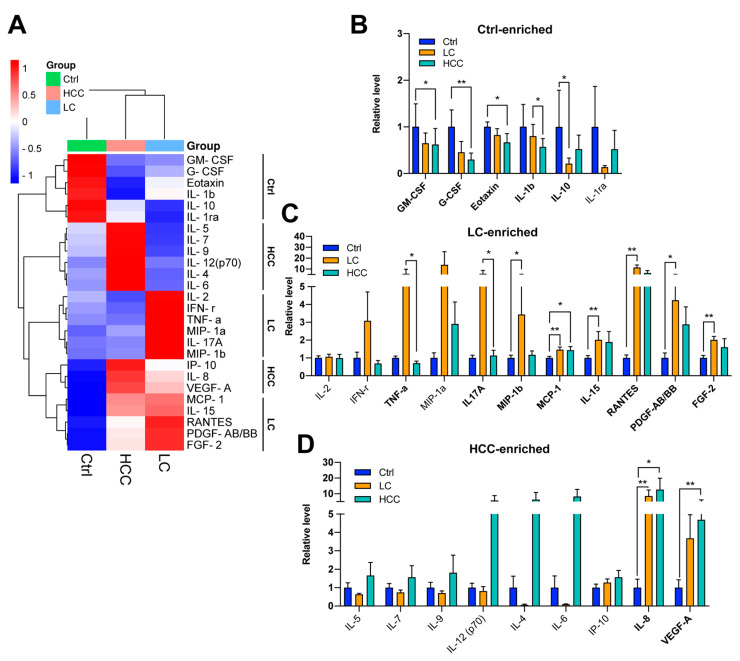
Changes in plasma cytokines/chemokines in patients with LC and HCC. (**A**) Mean levels of selected plasma cytokines/chemokines in the three cohorts are analyzed using heatmap. Comparison of plasma cytokine/chemokine levels between cohorts for candidates enriched in (**B**) the control cohort, (**C**) the LC cohort, and (**D**) the HCC cohort. The comparisons were made using the Kruskal–Wallis test for three groups and the post hoc Dunn’s test for two groups. *, *p* < 0.05; **, *p* < 0.01.

It is well known that dysbiosis in the gut or leaky gut can lead to endotoxemia, as shown in Figure 3A,B. Increased plasma endotoxin levels also correlate with an induced inflammatory response associated with increased levels of specific circulating cytokines/chemokines [32]. To understand whether cytokine/chemokine levels were altered in recruited subjects, the plasma levels of a panel of cytokines/chemokines were measured. The heatmap analysis of mean plasma cytokine/chemokine levels is presented in Figure 4A. All these plasma cytokines/chemokines were classified into three groups, which were enriched in the control, LC, and HCC cohorts, respectively. Further examination of the statistical significance between two of these three cohorts showed that GM-CSF, G-CSF, eotaxin, IL-1b, and IL-10 were enriched in the control cohort (Figure 4B); TNF-a, IL17A, MIP-1b, IL-15, RANTES, PDGF-AB/BB, and FGF-2 were enriched in the LC group (Figure 4C); and IL-8 and VEGF-A were enriched in the HCC cohort (Figure 4D). Details of the plasma cytokine/chemokine comparison are listed in Table 3.

### 3.7. Joint Correlation Analysis of Gut Microbiota, Plasma Metabolite, and Plasma Cytokine/Chemokine Signatures in All Subjects

**Figure 5:**
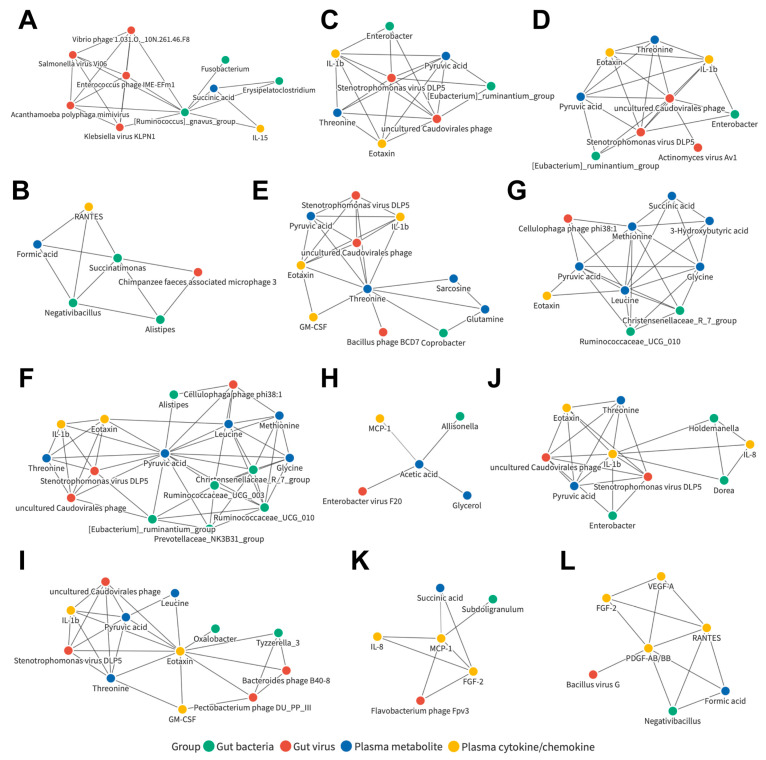
Correlation networks for candidates in all subjects. Detailed correlation network centered on the bacterial genera (**A**) *Ruminococcus gnavus group* and (**B**) *Succinatimonas*; the viral species (**C**) Stenotrophomonas virus DLP5 and (**D**) uncultured Caudovirales phage; the plasma metabolites (**E**) threonine, (**F**) pyruvic acid, (**G**) leucine, and (**H**) acetic acid; and the plasma cytokines/chemokines (**I**) eotaxin, (**J**) IL-1b, (**K**) MCP-1, and (**L**) PDGF-AB/BB.

**Figure 6:**
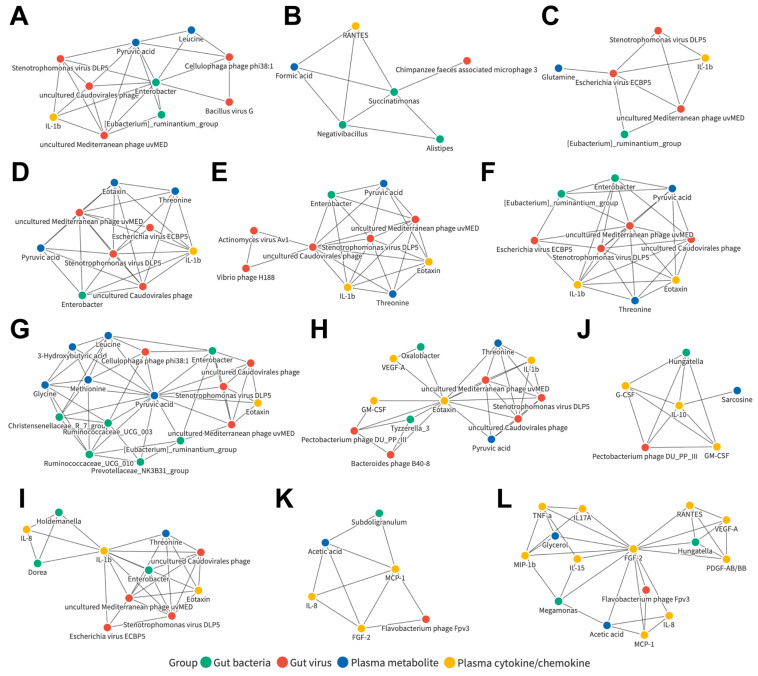
Correlation networks of candidates in the control and LC cohorts. Detailed correlation networks centered on the bacterial genera (**A**) *Enterobacter* and (**B**) *Succinatimonas*; the viral species (**C**) Escherichia virus ECBP5, (**D**) Stenotrophomonas virus DLP5, (**E**) uncultured Caudovirales phage, and (**F**) uncultured Mediterranean phage uvMED; the plasma metabolites (**G**) pyruvic acid; and the plasma cytokines/chemokines (**H**) eotaxin, (**I**) IL-1b, (**J**) IL-10, (**K**) MCP-1, and (**L**) FGF-2.

Based on the results shown in Figure 1, Figure 2, Figure 3 and Figure 4, a list of candidates with significant differences in gut bacterial and viral composition, as well as plasma aqueous phase metabolites and plasma cytokines/chemokines, was identified. Joint correlation analysis was performed encompassing all candidate markers and all recruited subjects (Figure S5). To understand the detailed relationships between the candidates, a correlation network was conducted (Figure S6). Twelve of these networks caught our attention because they were made up of components from four datasets, including those centered on the bacterial genera *Ruminococcus gnavus group* (Figure 5A) and *Succinatimonas* (Figure 5B); the viral species Stenotrophomonas virus DLP5 (Figure 5C) and uncultured Caudovirales phage (Figure 5D); the plasma metabolites threonine (Figure 6E), pyruvic acid (Figure 5F), leucine (Figure 5G), and acetic acid (Figure 5H); and the plasma cytokines/chemokines eotaxin (Figure 5I), IL-1b (Figure 5J), MCP-1 (Figure 5K), and PDGF-AB/BB (Figure 5L). This suggests that these correlated networks may be a potential common signature of LC and HCC patients.

### 3.8. Joint Correlation Analysis of Gut Microbiota, Plasma Metabolite, and Plasma Cytokine/Chemokine Signatures in the Control and LC Cohorts

To understand whether these associated networks could indeed be applied to LC patients as potential common characteristics, a similar joint analysis was performed when only the control and LC cohorts were included (Figure S7). Further investigations on the detailed relationships between the candidates in the related network were conducted (Figure S8). Among them, 12 networks were observed, consisting of components from four datasets, including those centered on the bacterial genera *Enterobacter* (Figure 6A) and *Succinatimonas* (Figure 6B); the viral species Escherichia virus ECBP5 (Figure 6C), Stenotrophomonas virus DLP5 (Figure 6D), uncultured Caudovirales phage (Figure 6E), and uncultured Mediterranean phage uvMED (Figure 6F); the plasma metabolites pyruvic acid (Figure 6G); and the plasma cytokines/chemokines eotaxin (Figure 6H), IL-1b (Figure 6I), IL-10 (Figure 6J), MCP-1 (Figure 6K), and FGF-2 (Figure 6L).

### 3.9. Joint Correlation Analysis of Gut Microbiota, Plasma Metabolite, and Plasma Cytokine/Chemokine Signatures in the Control and HCC Subjects

**Figure 7:**
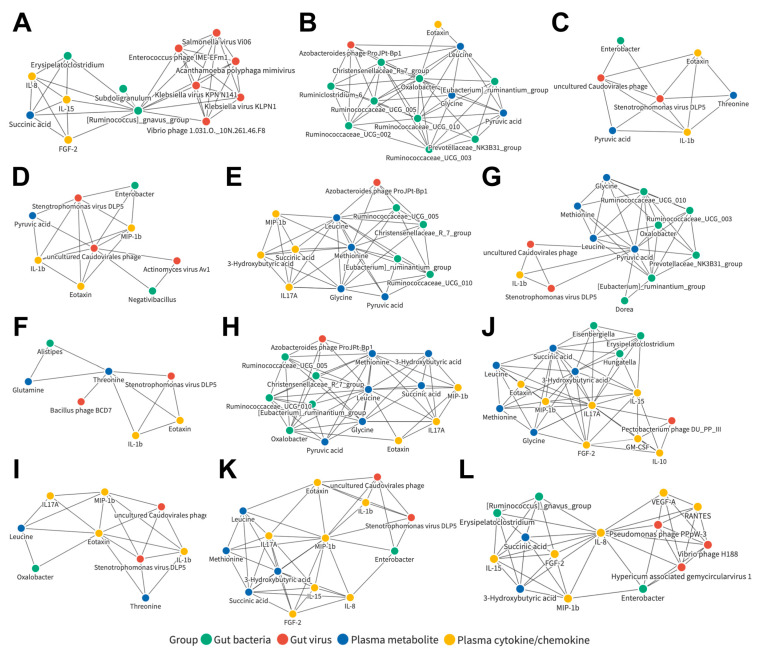
Correlation networks of candidates in the control and HCC subjects. Detailed correlation networks centered on the bacterial genera (**A**) *Ruminococcus gnavus group* and (**B**) *Oxalobacter*; the viral species (**C**) Stenotrophomonas virus DLP5 and (**D**) uncultured Caudovirales phage; the plasma metabolites (**E**) methionine, (**F**) threonine, (**G**) pyruvic acid, and (**H**) leucine; and the plasma cytokines/chemokines (**I**) eotaxin, (**J**) IL-17A, (**K**) MIP-1b, and (**L**) IL-8.

To understand whether these correlated networks could also be applied as potential common characteristics in HCC patients, a similar joint analysis was performed when only the control and HCC cohorts were included (Figure S9). Further investigations on the detailed relationships between the candidates in the relevant network were carried out (Figure S10). Of these, 12 networks were observed, consisting of components from four datasets, including those centered on the bacterial genera *Ruminococcus gnavus group* (Figure 7A) and *Oxalobacter* (Figure 7B); the viral species Stenotrophomonas virus DLP5 (Figure 7C) and uncultured Caudovirales phage (Figure 7D); the plasma metabolites methionine (Figure 7E), threonine (Figure 7F), pyruvic acid (Figure 7G), and leucine (Figure 7H); and the plasma cytokines/chemokines eotaxin (Figure 7I), IL-17A (Figure 7J), MIP-1b (Figure 7K), and IL-8 (Figure 7L).

### 3.10. Joint Correlation Analysis of Gut Microbiota, Plasma Metabolite, and Plasma Cytokine/Chemokine Signatures in the LC and HCC Subjects

**Figure 8:**
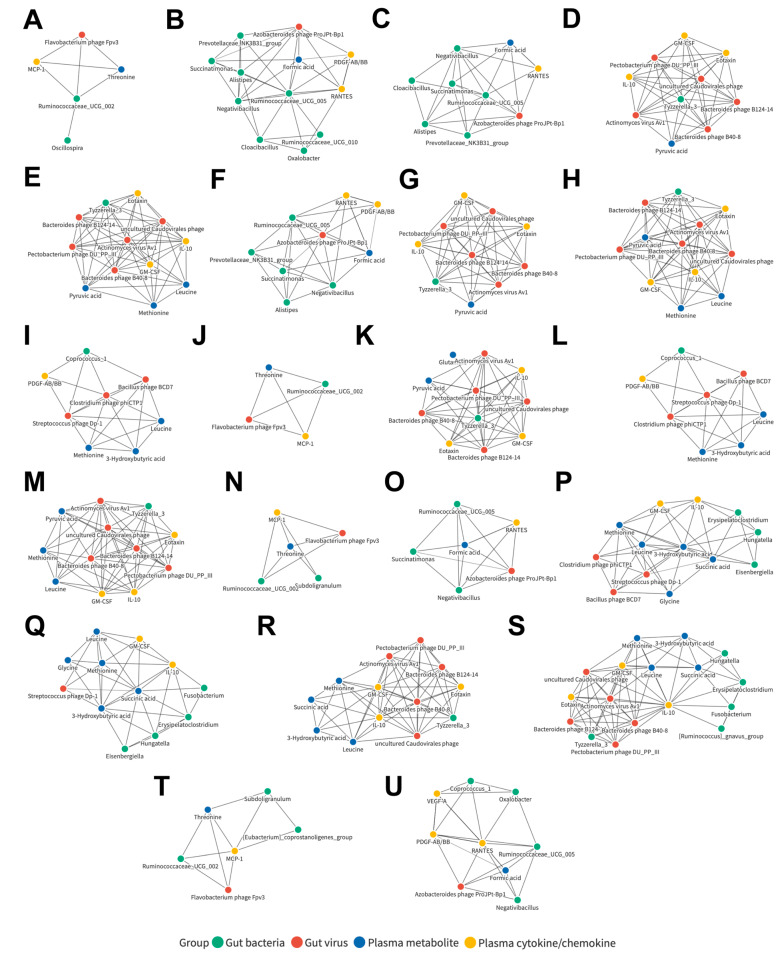
Correlation networks of candidates in the control and HCC subjects. Detailed correlation networks centered on the bacterial genera (**A**) *Ruminococcaceae UCG 002*, (**B**) *Ruminococcaceae UCG 005*, (**C**) *Negativibacillus*, and (**D**) *Tyzzerella 3;* the viral species (**E**) Actinomyces virus Av1, (**F**) Azobacteroides phage ProJPt-Bp1, (**G**) Bacteroides phage B124-14, (**H**) Bacteroides phage B40-8, (**I**) Clostridium phage phiCTP1, (**J**) Flavobacterium phage Fpv3, (**K**) Pectobacterium phage DU_PP_III, (**L**) Streptococcus phage Dp-1, and (**M**) uncultured Caudovirales phage; the plasma metabolites (**N**) threonine, (**O**) formic acid, (**P**) 3-hydroxybutyric acid, and (**Q**) Succinic acid; and the plasma cytokines/chemokines (**R**) GM-CSF, (**S**) IL-10, (**T**) MCP-1, and (**U**) RANTES.

Finally, to understand whether these associated networks could also be biomarkers correlated with liver disease severity, a similar joint analysis was performed when only the LC and HCC cohorts were included (Figure S11). Further investigation of the detailed relationships between candidates in the related network was conducted (Figure S12). Among them, 21 networks were identified, consisting of components from four datasets, including those of the bacterial genera *Ruminococcaceae UCG 002* (Figure 8A), *Ruminococcaceae UCG 005* (Figure 8B), *Negativibacillus* (Figure 8C), and *Tyzzerella 3* (Figure 8D); the viral species Actinomyces virus Av1 (Figure 8E), Azobacteroides phage ProJPt-Bp1 (Figure 8F), Bacteroides phage B124-14 (Figure 8G), Bacteroides phage B40-8 (Figure 8H), Clostridium phage phiCTP1 (Figure 8I), Flavobacterium phage Fpv3 (Figure 8J), Pectobacterium phage DU_PP_III (Figure 8K), Streptococcus phage Dp-1 (Figure 8L), and uncultured Caudovirales phage (Figure 8M); the plasma metabolites threonine (Figure 8N), formic acid (Figure 8O), 3-hydroxybutyric acid (Figure 8P), and Succinic acid (Figure 8Q); and the plasma cytokines/chemokines GM-CSF (Figure 8R), IL-10 (Figure 8S), MCP-1 (Figure 8T), and RANTES (Figure 8U).

### 3.11. Identification of Liver Disease Severity-Associated, LC or HCC Exclusive, or Common Biomarker Networks

A summary of the network centers identified in each subgroup is shown in Table 4. It also indicates whether specific networks are seen only in particular diseases, or whether specific networks may exist as a common characteristic in LC and HCC patients. Additionally, the center of networks associated with liver disease severity is also summarized. For networks centered on gut bacterial genera, they were found to be disease-specific and disease-severity-associated biomarker networks. Specifically, the *Ruminococcus gnavus group* and *Oxalobacter* were specific for HCC, while the *Succinatimonas* and *Enterobacter* were specific for LC. *Ruminococcaceae UCG 002, Ruminococcaceae UCG 005, Negativibacillus,* and *Tyzzerella 3* were biomarkers associated with liver disease severity.

Interestingly, two gut virus-centric networks, Stenotrophomonas virus DLP5 and uncultured Caudovirales phage, were common signatures in LC and HCC patients, whereas Escherichia virus ECBP5 and uncultured Mediterranean phage uvMED were specific for LC. Of note, uncultured Caudovirales phage may also be a biomarker of liver disease severity. In addition to uncultured Caudovirales phage, Actinomyces virus Av1, Azobacteroides phage ProJPt-Bp1, Bacteroides phage B124-14, Bacteroides phage B40-8, Clostridium phage phiCTP1, Flavobacterium phage Fpv3, Pectobacterium phage DU_PP_III, and Streptococcus phage Dp-1 were also served biomarkers for liver disease severity.

It was found that metabolite reprogramming may play a crucial role in HCC as the plasma metabolite-centric networks were most often identified as HCC-specific biomarkers, including threonine, leucine, and methionine. Only pyruvic-acid- and acetic-acid-centric networks were common signatures of LC and HCC patients. In particular, threonine was also found to be a biomarker for liver disease severity. In addition to threonine, formic acid, 3-hydroxybutyric acid, and succinic acid were also identified as centers of networks associated with liver disease severity.

Finally, two plasma cytokines/chemokines, including eotaxin and PDGF-AB/BB, were found to be common features in LC and HCC patients. IL-1b, MCP-1, IL-10, and FGF-2 were specific for LC, while IL-17A, MIP-1b, and IL-8 were specific for HCC. Notably, MCP-1 and IL-10 were also found to be the centers of networks associated with liver disease severity. In addition to them, GM-CSF and RANTEE were also identified as potent biomarkers of liver disease severity. This finding suggests that these networks may be biomarkers of LC and HCC separately or simultaneously. Additionally, they may also serve as biomarkers of the severity of liver disease.

## 4. Discussion

This study attempted to use multi-omics tools to identify the exclusive or common disease signatures or biomarkers of liver disease severity, consisting of gut microbiota (bacteria and viruses), plasma metabolites, and plasma cytokines/chemokines, which may be a contributing factor in LC and HCC, beyond the known etiologies. Here, we showed that the gut dysbiosis of specific bacteria and viruses appeared in the LC and HCC groups, although the diversity did not differ significantly between the groups (Figure 1 and Figure 2). The plasma levels of zonulin, a biomarker of an impaired intestinal barrier, and bacterial LPS further suggest that leaky gut and endotoxemia states are associated with liver disease severity (Figure 3A–C). Although plasma TMAO levels did not differ significantly between groups, differences in the metabolic reprogramming of plasma metabolites were observed between groups (Figure 3D–H). Additionally, a different cytokine/chemokine profile was, respectively, observed in LC and HCC patients compared to controls (Figure 4). Joint correlations and network analyses further revealed a list of disease-specific and common signatures shared by patients with LC or HCC, as well as biomarkers associated with liver disease severity (Figure 5, Figure 6, Figure 7, Figure 8 and Figures S5–S12 and Table 4).

In this study, the HCC group was significantly older in age than the Ctrl group (Table 1), possibly due to the higher incidence of HCC occurring in Taiwanese elderly after more than 30 years of routine HBV vaccination (36% reduction in HCC incidence for those <30 years of age) and a 15-year national viral therapy program (15% reduction in HCC incidence between ages 30 and 69) [35]. Although age may be a major determinant of human gut microbial diversity in the US, the UK, and Columbia, no association between alpha diversity and age was observed in the Chinese cohort [36]. Another notable characteristic was BMI, which was significantly higher in the LC group compared to controls (Table 1). High BMI is now considered to be a well-established risk factor for LC, regardless of etiology (HBV, HCV, or NAFLD) [37], implying that gut dysbiosis of bacteria and viruses may play a tributary role in the making of both obesity and LC.

Correlation network analysis found that the gut bacterial genera acted only as exclusive signatures for either LC or HCC, but not common characteristics (Table 4). In particular, networks centered on the *Ruminococcus gnavus group* and *Oxalobacter* were specific for HCC, whereas *Succinatimonas and Enterobacter* were specific for LC. In particular, in HCC patients, the *Ruminococcus gnavus group* increased and the *Oxalobacter* decreased. *Succinatimonas* elevated, and *Enterobacter* reduced only in the LC cohort (Figure 1F–H). The roles of most of these gut bacterial genera are yet to be defined in LC or HCC. An exception was consistently seen in the increased abundance of the *Ruminococcus gnavus group* of HCC [16]. Additionally, the *Ruminococcus gnavus group* has been shown to be enriched in the HCC tumor region, particularly in patients with chronic HBV or HCV infection [38]. On the other hand, networks centered on *Ruminococcaceae UCG 002, Ruminococcaceae UCG 005, Negativibacillus*, and *Tyzzerella 3* were found to be potent biomarkers of liver disease severity (Table 4). The abundance of *Ruminococcaceae UCG 002* and *Ruminococcaceae UCG 005* gradually decreased in the LC and HCC cohorts (Figure 1F), whereas *Negativibacillus* and *Tyzzerella 3* were both higher in the LC but significantly decreased in the HCC cohort (Figure 1G). This is consistent with the previous study showing that the intestinal bacterial family *Ruminococcaceae*, which includes the genera *Ruminococcaceae UCG 002* and *Ruminococcaceae UCG 005,* is present at a lower abundance in patients with severer liver disease [39]. This evidence suggests that they may indeed act as biomarkers of liver disease severity, particularly in the HCC cohort, where their abundances are lower.

Gut bacteriophage abundance has been implicated as an important factor in modulating gut bacterial composition, which may contribute to disease and cancer [40]. Here, correlation networks centered on two gut viral species, Stenotrophomonas virus DLP5 and uncultured Caudovirales phage, which were identified as common signatures in LC and HCC patients (Table 4). Neither of these two viruses was present in the LC and HCC cohorts (Figure 2G). This may be the reason why networks around them are common characteristics in both LC and HCC patients. The decreased abundance of Stenotrophomonas virus DLP5 is expected to increase the abundance of its host bacterium, *Stenotrophomonas*. However, no OTUs belonging to the bacterial genus *Stenotrophomonas* were found in this study (Table S1). Nevertheless, pathogens belonging to the bacterial genus *Stenotrophomonas*, such as *Stenotrophomonas maltophilia*, have been found to be enriched in the microbiota of HCC tumors in association with the risk of LC and HCC progression [41]. The presence of *Stenotrophomonas maltophilia* in the liver may be a combined consequence of the reduced abundance of gut Stenotrophomonas virus DLP5 and the leaky gut, commonly seen in LC and HCC patients (Figure 3B). Additionally, the Escherichia virus ECBP5 and the uncultured Mediterranean phage uvMED are central to the LC-specific networks, mainly because they are absent in the LC cohort (Figure 2G). On the other hand, uncultured Caudovirales phage, Actinomyces virus Av1, Azobacteroides phage ProJPt-Bp1, Bacteroides phage B124-14, Bacteroides phage B40-8, Clostridium phage phiCTP1, Flavobacterium phage Fpv3, Pectobacterium phage DU_PP_III, and Streptococcus phage Dp-1 were found to be biomarkers of liver disease severity. Except for Bacteroides phage B124-14, Bacteroides phage B40-8, Flavobacterium phage Fpv3, and Pectobacterium phage DU_PP_III, which were high in the LC but significantly decreased in the HCC cohort, the rest gradually decreased (Figure 2G). This suggests that they could indeed serve as biomarkers of liver disease severity, especially for those who were progressively declining in the LC and HCC cohorts. However, because the hosts of most identified viruses are unknown, their potential roles in the pathogenesis of LC and HCC remain elusive and need to be clarified.

TMAO was previously shown to be an important metabolite derived from choline, depending on the composition of the gut microbiota [42]. However, in this study, we found that TMAO levels did not differ significantly between groups, even in the presence of marked endotoxemia and increased gut barrier permeability (Figure 3A–D). Correlation network analysis found that only pyruvic acid and acetic-acid-centered networks were common signatures of LC and HCC patients (Table 4). In this study, it was found that plasma concentrations of pyruvic acid reduced gradually, whereas acetic acid increased gradually in LC and HCC patients (Figure 3F–H), although their levels in LC and HCC patients have been debated [16,43,44,45]. On the other hand, three metabolites were identified as biomarkers of liver disease severity, including formic acid, 3-hydroxybutyric acid, and succinic acid (Table 4). Formic acid may be selected as a possible biomarker of liver disease severity simply because it was dramatically increased in the LC group but not in the HCC group (Figure 3G), thus suggesting its involvement in the disease process. As for 3-hydroxybutyric acid and succinic acid, there was no significant difference between the LC group and the control group. However, they were all significantly increased in the HCC group (Figure 3H), and, therefore, considered biomarkers of liver disease severity. In line with previous studies, for unknown reasons, it has been observed that gut dysbiosis is associated with the enriched circulating or fecal levels of acetic acid, succinic acid, and formic acid, which are short-chain fatty acids or their precursors with anti-inflammatory effects, in patients with NAFLD-related LC or HCC [7,9].

Intestinal barrier impairment due to gut dysbiosis was evidenced by increased zonulin and endotoxemia in the LC and HCC groups. It activates a series of inflammatory pathways involving interleukins and other cytokines, resulting in locoregional effects, such as gut and liver inflammation or a systemic inflammatory response mediated by microbial structural elements [46]. Correlation networks centered around eotaxin and PDGF-AB/BB were found to be common features of LC and HCC patients. Eotaxin decreased with progressive liver disease, but PDGF-AB/BB increased in LC and HCC patients (Figure 4B,C). The role of eotaxin in HCC is unclear. In other cancers, it is thought to be beneficial for anti-tumor effects by attracting eosinophils [47,48]. In contrast, elevated PDGF-AB/BB has been reported to be associated with the severity of liver diseases [49]. Correlation networks centered on plasma cytokines/chemokines, IL-1b, MCP-1, IL-10, and FGF-2, were observed to be specific for LC. It has been reported that these cytokines/chemokines can induce persistent liver inflammation, fibrosis, and LC associated with alcoholic, nonalcoholic, or virus-related hepatic injury [50,51,52]. In contrast, IL-10, the only cytokine whose levels were reduced in the LC group, is known for its role in controlling anti-inflammatory effects [53]. We found that IL-17A-, MIP-1b-, and IL-8-centric correlation networks were specifically observed in HCC. These cytokines/chemokines have been reported to promote hepatocarcinogenesis or progression by enhancing HCC cell growth or migration, constitutively activating inflammatory pathways, or promoting angiogenesis [54,55,56].

Although there are few differences in the composition of gut bacteria and viruses, plasma metabolites, and cytokines/chemokines between this study and previous studies, this study shows a high level of consistency compared to previous studies. These pre-existing differences may be due to differences in patient recruitment between the current study and previous studies, such as etiology, age, gender, and eating habits, and this may also have an influence on the authenticity of findings in this study. Despite existing limitations, this study confirmed the gut microbial and viral compositional differences from healthy controls to that in patients with LC and HCC with disease severity signatures, further influencing host metabolic reprogramming and cytokine/chemokine skewness, which, without a doubt will affect the hepatic microenvironment to facilitate HCC development. This pilot study has broadened the potential therapeutic targets in LC or even HCC by manipulating the components of the deregulated gut–liver axis in the future.

## 5. Conclusions

Gut dysbiosis is differentially associated with LC and HCC and is associated with an impaired gut barrier, accompanied by altered systemic cytokine/chemokine profiles, and metabolic products. A list of common network signatures shared between LC and HCC patients center on the gut virus Stenotrophomonas virus DLP5 and uncultured Caudovirales phage, the plasma metabolites pyruvic acid and acetic acid, and the plasma cytokines/chemokines eotaxin and PDGF-AB/BB, regardless of etiology. Additionally, network features unique to LC or HCC were also identified, including the enterobacterial genera *Succinatimonas* and *Enterobacter*; the enterovirus Escherichia virus ECBP5 and uncultured Mediterranean phage uvMED; the plasma cytokines/chemokines IL-1b, MCP-1, IL-10, and FGF-2 for LC; the gut bacterial genera *Ruminococcus gnavus group* and *Oxalobacter*; the plasma metabolites threonine, leucine, and methionine; and the plasma cytokines/chemokines IL-17A, MIP-1b, and IL-8 for HCC. This multi-omics study provides novel insights that altered gut microbial/viral composition associated with the LC of various etiologies may result in metabolic reprogramming and transforming immunological microenvironment to facilitate hepatocarcinogenesis.

## Figures and Tables

**Table 1:** Comparison of characteristics between study cohorts.

Parameter	Ctrl (*n* = 17) ^†^	LC (*n* = 18) ^†^	HCC (*n* = 10) ^†^	P_Ctrl-LC_^‡^	P_Ctrl-HCC_^‡^	P_LC-HCC_^‡^
Age (Year)	56.7 ± 9.8	65.2 ± 11.4	70.2 ± 5.0	**0.024**	**<0.001**	0.202
Sex (Male:Female)	7:10	12:6	6:4	0.241	0.585	0.724
BMI	23.1 ± 1.51	26.2 ± 3.6	25.0 ± 4.1	**0.002**	0.094	0.480
Etiology(B:C:other)	-	11:4:3	6:4:0	-	-	0.304
AST (U/L)	30.9 ± 3.5	35.6 ± 10.8	43.3 ± 17.1	0.097	**0.007**	0.155
ALT (U/L)	28.5 ± 4.7	30.0 ± 17.9	48.1 ± 30.0	0.740	**0.013**	0.055
Bilirubin (mg/dL)	-	1.1 ± 0.5	1.2 ± 0.7	-	-	0.664
Albumin (g/dL)	-	4.4 ± 0.4	3.6 ± 0.5	-	-	**<0.001**
T-Cholesterol (mg/dL)	-	166.6 ± 26.1	150.0 ± 40.3	-	-	0.196
Triglyceride (mg/dL)	-	108.6 ± 54.8	99.4 ± 59.1	-	-	0.682
HbA1c (%)	-	6.1 ± 1.2	6.1 ± 0.9	-	-	0.999
AFP (ng/mL)	-	10.9 (1.0–25.1)	101.8 (1.0–250.5)	-	-	0.075
Platelet (1000/μL)	-	120.2 ± 45.8	100.7 ± 43.6	-	-	0.283
NAs treatment (Yes:No)		10:8	6:4			0.820
DAA treatment (Yes:No)		4:14	4:6			0.575
Child-Pugh score		5.1 ± 0.2	6.0 ± 1.6			0.158

^†^ Parametric data are presented as mean ± standard deviation, whereas for non-normally distributed data, they are presented as median (range); ^‡^ *p* values were obtained using Student’s *t*-test when data were normally distributed, while the Mann–Whitney test was used for data that were not normally distributed; Dichotomized data are presented as numbers and percentages (%) and compared using the chi-square test; Bold values indicate statistical significance *p* < 0.05; Ctrl, control group; LC, liver cirrhosis group; HCC, hepatocellular carcinoma group; BMI, body mass index (kg/m^2^); B, chronic hepatitis B virus infection, C, chronic hepatitis C virus infection, other, other etiologies; AST, aspartate transaminase; ALT, alanine transaminase; T-Cholesterol, total Cholesterol; HbA1c, hemoglobin A1c; AFP, Alpha-fetoprotein; NA, nucleotide analog (anti-HBV treatment); DAA, direct-acting antiviral (anti-HCV agent).

**Table 2:** Plasma aqueous metabolite levels in the healthy control, cirrhosis, and HCC cohorts.

Metabolite	Ctrl (*n* = 17) ^†^	LC (*n* = 18) ^†^	HCC (*n* = 10) ^†^	P_Ctrl-LC_^‡^	P_Ctrl-HCC_^‡^	P_LC-HCC_^‡^
Ethanol, μM	82.4 ± 95.4	35.1 ± 112.8	158.0 ± 205.1	0.168	0.830	0.189
Trimethylamine-N-oxide, μM	26.5 ± 17.9	28.3 ± 19.0	32.6 ± 14.3	0.975	0.393	0.402
2-Aminobutyric acid, μM	46.9 ± 64.7	17.7 ± 27.6	58.8 ± 130.3	0.179	0.401	0.824
Alanine, mM	19.7 ± 32.5	9.6 ± 21.4	15.7 ± 32.9	0.587	0.574	0.892
Creatine, μM	43.5 ± 26.5	45.4 ± 106.8	21.5 ± 21.6	0.072	0.086	0.898
Creatinine, μM	58.0 ± 35.2	66.0 ± 36.7	64.9 ± 36.2	0.560	0.582	0.927
Glutamic acid, mM	26.0 ± 41.3	15.5 ± 34.7	23.4 ± 42.4	0.103	**0.027**	0.348
Glutamine, μM	336.4 ± 238.9	32.9 ± 125.6	0.0 ± 0.0	**<0.001**	**<0.001**	0.617
Glycine, μM	372.6 ± 259.2	219.2 ± 164.9	278.1 ± 154.3	**0.022**	0.594	0.197
Histidine, μM	63.9 ± 47.8	49.4 ± 33.3	44.5 ± 42.1	0.354	0.329	0.804
Isoleucine, μM	40.3 ± 38.6	876.1 ± 3525.0	38.0 ± 29.1	0.171	0.755	0.438
Leucine, μM	203.5 ± 135.8	141.2 ± 131.4	165.3 ± 129.9	**0.027**	0.346	0.416
Lysine, μM	98.3 ± 93.2	160.0 ± 222.1	200.4 ± 163.8	0.722	0.129	0.212
Methionine, μM	150.4 ± 162.3	89.3 ± 155.0	94.5 ± 161.4	**0.008**	**0.003**	0.397
N,N-Dimethylglycine, mM	29.4 ± 47.0	16.7 ± 38.4	25.0 ± 46.3	0.200	0.136	0.627
Ornithine, μM	28.1 ± 42.5	80.3 ± 86.8	68.9 ± 65.4	0.092	0.180	0.991
Phenylalanine, mM	1.5 ± 2.3	0.7 ± 1.4	1.0 ± 1.7	0.683	0.584	0.380
Proline, μM	13.0 ± 53.6	36.2 ± 86.0	0.0 ± 0.0	0.291	0.633	0.186
Sarcosine, μM	3.8 ± 3.4	1.3 ± 1.5	1.6 ± 2.3	**0.032**	0.101	0.957
Threonine, μM	40.0 ± 54.8	4.9 ± 12.7	4.9 ± 7.2	**0.038**	0.261	0.608
Tyrosine, μM	37.7 ± 28.2	40.7 ± 23.8	43.1 ± 26.4	0.967	0.691	0.664
Valine, μM	181.6 ± 121.4	190.7 ± 86.7	146.8 ± 96.6	0.804	0.318	0.417
2-Hydroxybutyric acid, mM	29.2 ± 46.6	14.6 ± 34.1	22.0 ± 41.0	0.258	0.946	0.406
Acetic acid, μM	39.1 ± 34.9	150.8 ± 87.2	200.5 ± 94.2	**<0.001**	**<0.001**	0.343
Citric acid, mM	0.1 ± 0.1	1.3 ± 4.8	4.7 ± 8.7	0.891	0.785	0.701
Formic acid, μM	50.7 ± 28.2	148.6 ± 104.6	94.0 ± 45.1	**<0.001**	**0.035**	0.505
Lactic acid, mM	32.0 ± 44.8	18.2 ± 32.6	25.6 ± 38.9	0.449	0.948	0.591
Succinic acid, μM	45.5 ± 73.7	56.2 ± 86.2	158.1 ± 214.1	0.059	**0.008**	0.248
Choline, μM	168.9 ± 594.2	17.6 ± 33.8	37.1 ± 58.3	0.333	0.766	0.284
2-Oxoglutaric acid, μM	41.6 ± 74.5	46.6 ± 92.3	125.9 ± 231.3	0.991	0.791	0.796
**3-Hydroxybutyric acid**, mM	12.8 ± 22.6	9.1 ± 24.7	24.9 ± 45.8	0.143	**0.023**	0.257
Acetoacetic acid, μM	169.8 ± 271.3	61.4 ± 165.5	4.5 ± 3.6	0.714	0.478	0.672
Acetone, μM	26.8 ± 19.2	144.1 ± 451.8	544.3 ± 935.1	0.148	0.130	0.707
**Pyruvic acid**, μM	339.6 ± 206.6	95.5 ± 156.2	29.5 ± 21.0	**<0.001**	**<0.001**	0.333
D-Galactose, mM	28.9 ± 46.0	11.0 ± 31.9	0.0 ± 0.1	0.180	0.164	0.736
Glucose, mM	3.3 ± 2.8	3.3 ± 2.8	2.4 ± 2.2	0.962	0.555	0.578
**Glycerol**, μM	15.3 ± 24.1	40.6 ± 52.5	77.3 ± 97.4	**0.027**	**0.005**	0.272
Dimethylsulfone, μM	58.7 ± 98.0	32.4 ± 72.9	76.1 ± 138.9	0.948	0.829	0.867
**Ca-EDTA**, mM	30.6 ± 45.4	18.2 ± 37.7	25.7 ± 45.3	**0.012**	**0.013**	0.611
K-EDTA, mM	3.8 ± 2.6	3.5 ± 2.2	3.4 ± 4.0	0.237	0.222	0.771

^†^ Data are presented as mean ± standard deviation; Ctrl, control group; LC, liver cirrhosis group; HCC, hepatocellular carcinoma group; ^‡^ *p* values were obtained using the Kruskal–Wallis test for three groups and the post hoc Dunn’s test for two groups; A *p* <0.05 was considered significant; Those with statistical significance were labeled in bold.

**Table 3:** Plasma cytokine/chemokine profiles in the healthy control, cirrhosis, and HCC cohorts.

Cytokine/Chemokine	Ctrl (*n* = 17) ^†^	LC (*n* = 18) ^†^	HCC (*n* = 10) ^†^	P_Ctrl-LC_^‡^	P_Ctrl-HCC_^‡^	P_LC-HCC_^‡^
IL-1b (pg/mL)	2.4 ± 4.8	1.9 ± 2.6	1.4 ± 1.4	0.466	0.196	**0.048**
IL-1ra (ng/mL)	128.4 ± 458.2	17.1 ± 21.0	66.6 ± 165.5	0.857	0.348	0.426
IL-2 (pg/mL)	1.4 ± 0.7	1.4 ± 0.9	1.3 ± 1.0	0.587	0.121	0.270
IL-4 (pg/mL)	9.4 ± 24.1	0.5 ± 1.9	57.6 ± 135.5	0.971	0.249	0.257
IL-5 (pg/mL)	1.7 ± 1.9	1.1 ± 0.4	2.8 ± 3.9	0.306	0.700	0.625
IL-6 (pg/mL)	2.2 ± 5.9	0.2 ± 0.3	18.3 ± 32.3	0.474	0.592	0.248
IL-7 (pg/mL)	3.9 ± 3.7	2.9 ± 2.1	6.1 ± 7.9	0.494	0.436	0.842
IL-8 (pg/mL)	3.6 ± 6.9	30.6 ± 60.0	45.7 ± 82.8	**<0.001**	**0.018**	0.598
IL-9 (pg/mL)	0.5 ± 0.6	0.3 ± 0.3	0.9 ± 1.5	0.193	0.160	0.762
IL-10 (pg/mL)	3.2 ± 10.5	0.7 ± 1.7	1.7 ± 3.1	**0.016**	0.080	0.766
IL-12 (p70) (pg/mL)	2.0 ± 1.9	1.6 ± 2.1	11.0 ± 19.8	0.059	0.835	0.159
IL-15 (pg/mL)	1.5 ± 0.8	3.0 ± 2.9	2.8 ± 2.8	**0.006**	0.154	0.368
IL-17a (pg/mL)	1.6 ± 1.0	9.2 ± 22.5	1.9 ± 1.6	0.283	0.364	**0.048**
FGF-2 (pg/mL)	32.4 ± 19.2	65.0 ± 28.3	51.6 ± 50.7	**0.002**	0.4011	0.073
G-CSF (pg/mL)	4.7 ± 7.0	2.1 ± 4.6	1.4 ± 2.1	0.109	**0.006**	0.156
GM-CSF (pg/mL)	6.6 ± 13.4	4.3 ± 6.2	4.1 ± 7.2	0.277	**0.039**	0.250
IFN-γ(pg/mL)	3.7 ± 5.0	11.5 ± 25.7	2.6 ± 2.0	0.731	0.339	0.207
MCP-1 (pg/mL)	236.2 ± 85.3	343.7 ± 152.8	337.3 ± 160.0	**0.008**	**0.047**	0.690
MIP-1a (pg/mL)	2.7 ± 3.2	37.9 ± 140.1	7.9 ± 10.6	0.662	0.159	0.295
MIP-1b (pg/mL)	40.4 ± 25.3	138.8 ± 320.5	47.3 ± 28.4	**0.046**	0.669	0.258
Eotaxin (pg/mL)	76.7 ± 33.6	63.0 ± 45.3	51.1 ± 45.6	0.116	**0.046**	0.500
IP-10 (ng/mL)	0.6 ± 0.4	0.7 ± 0.5	0.9 ± 0.7	0.219	0.316	0.968
TNF-α (pg/mL)	9.6 ± 4.0	53.4 ± 174.2	6.8 ± 3.6	0.565	0.190	**0.049**
VEGF-a (pg/mL)	22.9 ± 40.5	84.1 ± 125.3	107.2 ± 105.8	0.064	**0.007**	0.626
PDGF-AB/BB (ng/mL)	17.9 ± 20.7	75.9 ± 73.1	51.6 ± 56.2	**0.015**	0.101	0.666
RANTES (ng/mL)	52.5 ± 35.9	595.2 ± 542.2	333.5 ± 364.8	**0.007**	0.435	0.123

^†^ Data are presented as mean ± standard deviation. Ctrl, control group; LC, liver cirrhosis group; HCC, hepatocellular carcinoma group; ^‡^ *p* values were obtained using the Kruskal–Wallis test for three groups and the post hoc Dunn’s test for two groups; A *p* < 0.05 was considered significant; Those with statistical significance were labeled in bold.

**Table 4:** Summary of biomarker networks identified in subjects.

Network Centered on	All Subjects	Ctrl-LC	Ctrl-HCC	Exclusive or Common Signature	Liver Disease Severity-Associated (LC-HCC)
*Ruminococcus gnavus group*	Yes	-	Yes	HCC specific	-
*Succinatimonas*	Yes	Yes	-	LC specific	-
*Enterobacter*	-	Yes	-	LC specific	-
*Oxalobacter*	-	-	Yes	HCC specific	-
*Ruminococcaceae UCG 002*	-	-	-	-	Yes
*Ruminococcaceae UCG 005*	-	-	-	-	Yes
*Negativibacillus*	-	-	-	-	Yes
*Tyzzerella 3*	-	-	-	-	Yes
Stenotrophomonas virus DLP5	Yes	Yes	Yes	Common	-
Uncultured Caudovirales phage	Yes	Yes	Yes	Common	Yes
Escherichia virus ECBP5	-	Yes	-	LC specific	-
Uncultured Mediterranean phage uvMED	-	Yes	-	LC specific	-
Actinomyces virus Av1	-	-	-	-	Yes
Azobacteroides phage ProJPt-Bp1	-	-	-	-	Yes
Bacteroides phage B124-14	-	-	-	-	Yes
Bacteroides phage B40-8	-	-	-	-	Yes
Clostridium phage phiCTP1	-	-	-	-	Yes
Flavobacterium phage Fpv3	-	-	-	-	Yes
Pectobacterium phage DU_PP_III	-	-	-	-	Yes
Streptococcus phage Dp-1	-	-	-	-	Yes
Threonine	Yes	-	Yes	HCC specific	Yes
pyruvic acid	Yes	Yes	Yes	Common	-
Leucine	Yes	-	Yes	HCC specific	-
Acetic acid	Yes	-	-	Common *	-
Methionine	-	-	Yes	HCC specific	-
Formic acid	-	-	-	-	Yes
3-hydroxybutyric acid	-	-	-	-	Yes
Succinic acid	-	-	-	-	Yes
Eotaxin	Yes	Yes	Yes	Common	-
IL-1b	Yes	Yes	-	LC specific	-
MCP-1	Yes	Yes	-	LC specific	Yes
PDGF-AB/BB	Yes	-	-	Common *	-
IL-10	-	Yes	-	LC specific	Yes
FGF-2	-	Yes	-	LC specific	-
IL-17A	-	-	Yes	HCC specific	-
MIP-1b	-	-	Yes	HCC specific	-
IL-8	-	-	Yes	HCC specific	-
GM-CSF	-	-	-	-	Yes
RANTES	-	-	-	-	Yes

* Only serving a common signature if all subjects were included; Plasma acetic acid and plasma PDGF-AB/BB levels were relatively lower in the control cohort compared to the LC and HCC groups and were, therefore, considered common characteristics of LC and HCC patients.

## Data Availability

All relevant data of this study are available within the article and its supplementary files or from the corresponding author on reasonable request.

## Supplementary Materials

The following supporting information can be downloaded at: https://www.mdpi.com/article/10.3390/cancers15010210/s1, Figure S1: Linear discriminant analysis (LDA) effect size (LEfSe) analysis identifies potential gut bacterial biomarkers for LC and HCC patients; Figure S2: Histogram of the LDA scores for differentially abundant bacterial taxon between groups; Figure S3: Linear discriminant analysis (LDA) effect size (LEfSe) analysis for identifying gut viral biomarkers of LC and HCC patients; Figure S4: Histogram of the LDA scores for differentially abundant viral taxon between groups; Figure S5: Joint correlation analysis when all subjects are included; Figure S6: Correlation networks of candidates in all subjects; Figure S7: Joint correlation analysis when including the control and LC cohorts; Figure S8: Correlation networks of candidates in the control and LC cohorts; Figure S9: Joint correlation analysis when including the control and HCC cohorts; Figure S10: Correlation networks of candidates in the control and HCC cohorts; Figure S11: Joint correlation analysis when including the LC and HCC cohorts; Figure S12: Correlation networks of candidates in the LC and HCC cohorts; Table S1: Abundance of gut bacterial genus in healthy control, cirrhosis and HCC cohorts; Table S2: Abundance of gut virus species in the healthy control, cirrhosis and HCC cohorts.
