# Supplementary material for: Multi-Omics Analyses Identify Signatures in Patients with Liver Cirrhosis and Hepatocellular Carcinoma
Source: Cancers (Basel). 2022 Dec 29;15(1):210. doi: 10.3390/cancers15010210 (PMC9818216; doi:10.3390/cancers15010210)
Supplement: Supplementary file 1 [file cancers-15-00210-s001.zip › cancers-2098827-supplementary.pdf]

Supplementary information

Supplementary Materials and Methods

Fecal DNA virus genome sequencing

For purification of virus-like particles, a 2-5 g aliquot of each stool sample was re-suspended in 25 mL of SM buffer [100 mM NaCl, 8 mM $MgSO_4$, 50 mM Tris (pH 7.5), and 0.002% gelatin (w/v)]. After centrifugation at 2500 × g for 10 min at room temperature, the resulting supernatant was removed and passed sequentially through 0.45 μm and 0.22 μm Whatman filters to remove residual cells. The filtrate was then adjusted to a density of 1.12 g/mL with CsCl and layered on top of a 3 mL step gradient prepared from 1 mL of CsCl solution at a density of 1.7 g/mL of SM buffer, 1.5 g/mL, and 1.35 g/mL. Samples were centrifuged at 60,000 × g at 4 °C for 2 h in an SW41 swinging bucket rotor (Beckman). The 1.5 g/mL layer was recovered since material in this density range is known to be enriched for bacteriophages.

After collecting the 1.5 g/mL layer, chloroform was added (0.2 volumes) and the mixture was centrifuged at 2500 × g for 5 min. To extract the virions, 0.5 mL of a solution containing 2 M Tris HCl (pH 8.5) and 0.2 M EDTA, 5 mL of formamide, and 1 μL of 20 mg/mL glycogen were added per 5 mL of sample, and the resulting mixture was incubated at room temperature for 30 min. After centrifugation for 20 min at 8000 × g at 4 °C, the pellet was washed twice with 70% ethanol. Finally, it was resuspended in 567 μL of TE buffer, followed by 30 μL of 10% SDS and 3 μL of a 20 mg/mL solution of Proteinase K (Fisher Scientific; cat no. AC61182-0500). The mixture was incubated at 55 °C for 1 h. Subsequent, 100 μL of 5 M NaCl and 80 μL of 10% cetyltrimethylammonium bromide were added. After a 10 min incubation at 65 °C, 700 μL of chloroform was added, and the mixture was centrifuged at 8000 × g for 5 min at room temperature. The resulting supernatant was transferred to a new tube and an equal volume of phenol/chloroform/isoamyl alcohol (25:24:1) was added, followed by centrifugation at 8000 × g for 5 min at room temperature. The supernatant was recovered, and an equal volume of chloroform was added. Following centrifugation, the supernatant was collected, and 0.7 volumes of isopropanol was used to precipitate the DNA. After re-centrifugation at 13,000 × g for 15 min at 4 °C, the pellet was washed with 500 μL of cold 70% ethanol, air-dried, and resuspended in 50 μL of TE buffer. An aliquot of purified DNA was used as a template for PCR with universal primers for the bacterial 16S rRNA and eukaryotic 18S rRNA genes to confirm the absence of non-viral DNA contamination.

Whole genome amplification was performed using Illustra™ GenomiPhi™ V2 kit (GE Healthcare) reagents and protocols to generate sufficient material for library construction. The amplified products were then pooled and cleaned using DNeasy kit (QIAGEN). A total of 1 μg of cleaned DNA was used for library construction using Illumina Nextera DNA Flex Library Prep (Illumina) according to manufacturer's instructions. The library was sequenced on the Illumina NovaSeq 6000 platform with paired reads of 150-bp in length.

The obtained raw data were transformed into raw sequenced reads using CASAVA base

calling and stored in FASTQ format. The resulting raw paired-end reads were filtered using Trimmomatic to remove low-quality reads, trim adaptor sequences, and eliminate poor-quality bases with the following parameters: LEADING:3 TRAILING:3 SLIDINGWINDOW:4:20 MINLEN:100 AVGQUAL:20 [21]. Cleaned reads were used to filter contaminating host sequences using Bowtie2 [22], assemble contigs using MEGAHIT [23], predict open reading frames using Prodigal [24], construct nonredundant gene catalog using CD-HIT [25], map to initial gene catalog using BWA [26], and create BAM files using SAMtools [27]. BAM file were used to calculate coverage of each sample using the MetaBAT2 pipeline [28]. Genome percentage completeness and contamination of all bins were assessed using CheckM [29]. The obtained unigenes were blast against the NCBI Refseq database using DIAMOND [30,31]. Taxonomic assignments were determined using the lowest common ancestor (LCA) algorithm. Gene annotation were conducted by aligning sequences across the multiple databases using DIAMOND, HMMER, and other annotators specified in the database.

Supplementary Figures

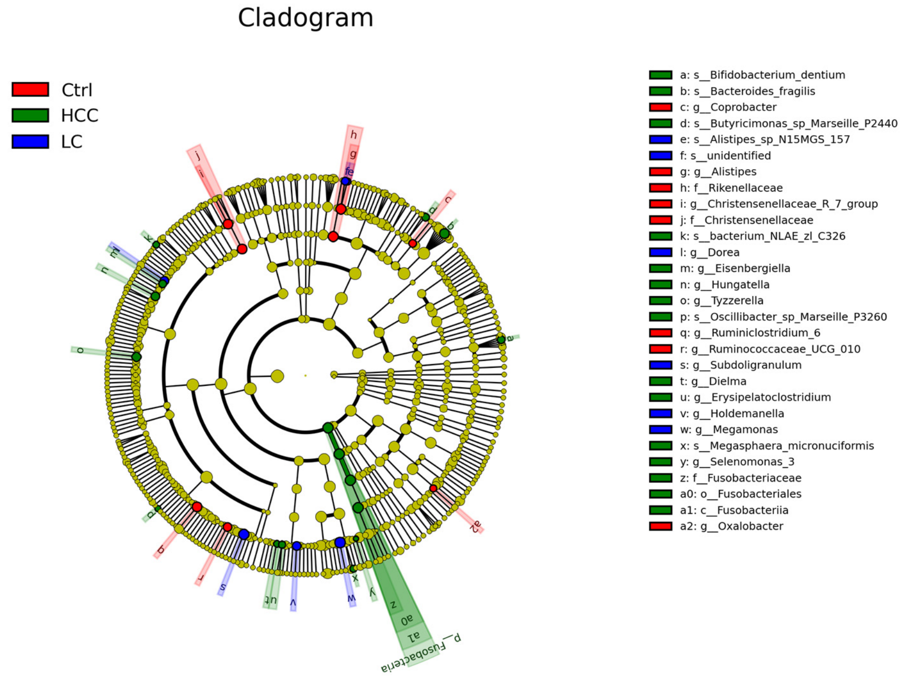

Figure S1. Linear discriminant analysis **(**LDA) effect size (LEfSe) analysis identifies potential gut bacterial biomarkers for LC and HCC patients. The cladogram shows differentially abundant taxonomic clades. Initials in the front of each taxonomic name are: c, class; o, order; f, family; g, genus; s, species.

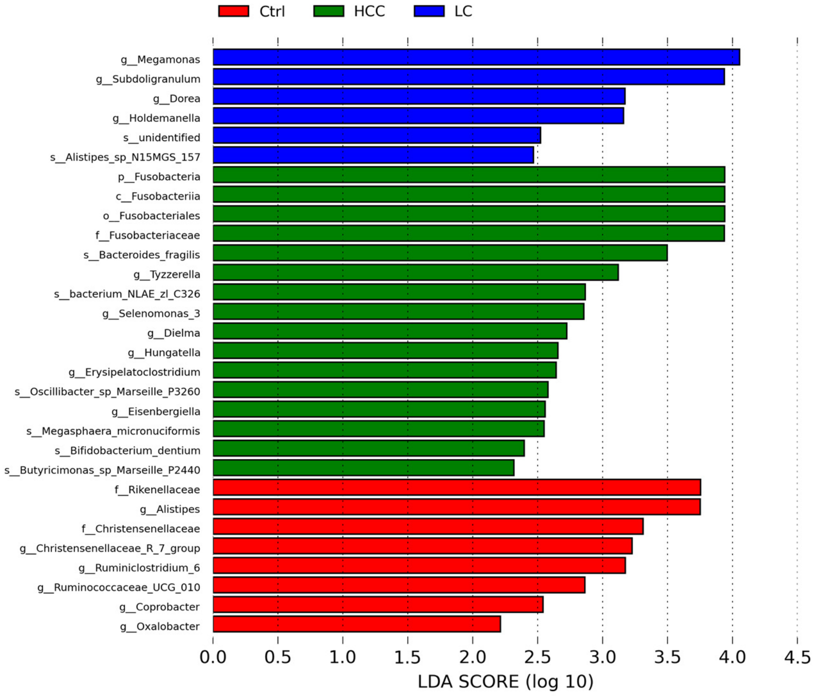

Figure S2. Histogram of the LDA scores for differentially abundant bacterial taxon between groups. Logarithmic LDA score higher than three indicates a higher relative abundance in the corresponding group than in the other group. Initials in the front of each taxonomic name are: p, phylum; c, class; o, order; f, family; g, genus; s, species.

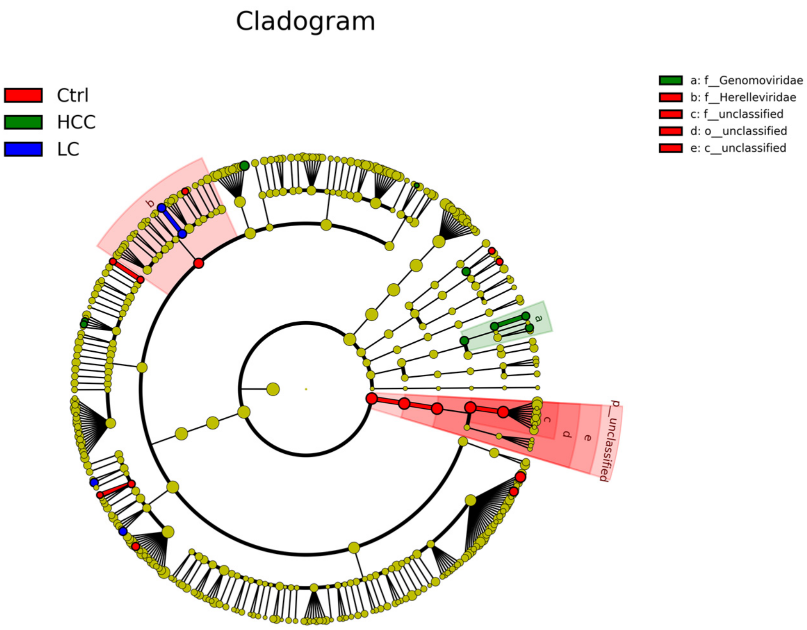

Figure S3. Linear discriminant analysis (LDA) effect size (LEfSe) analysis for identifying gut viral biomarkers of LC and HCC patients. The cladogram shows differentially abundant taxonomic clades. Initials in the front of each taxonomic name are: c, class; o, order; f, family.

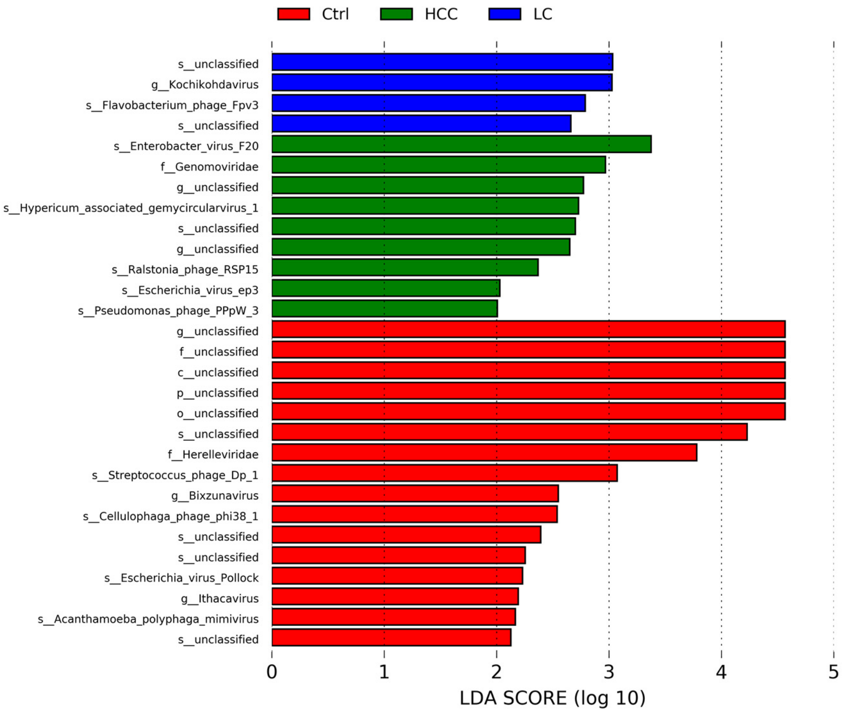

Figure S4. Histogram of the LDA scores for differentially abundant viral taxon between groups. Logarithmic LDA score higher than three indicates a higher relative abundance in the corresponding group than in the other group. Initials in the front of each taxonomic name are: p, phylum; c, class; o, order; f, family; g, genus; s, species.

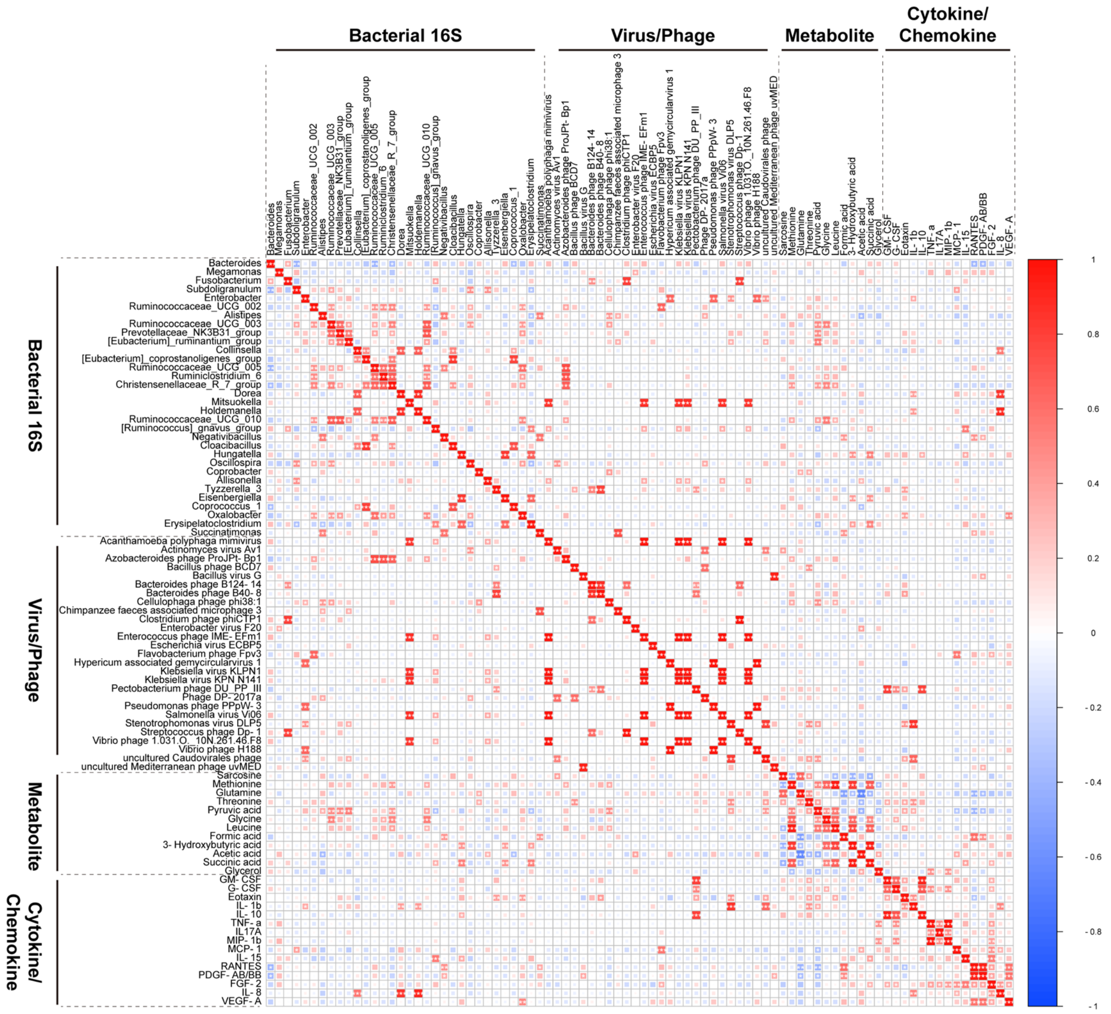

Figure S5. Joint correlation analysis when all subjects are included. Pearson correlation analysis of candidates from four data sets, including gut bacterial genus, gut viral species, plasma metabolite, and plasma cytokine/chemokine. The asterisk in the square indicates whether the correlation has a significant difference. *, P<0.05; **, P<0.01; ***, P<0.001.

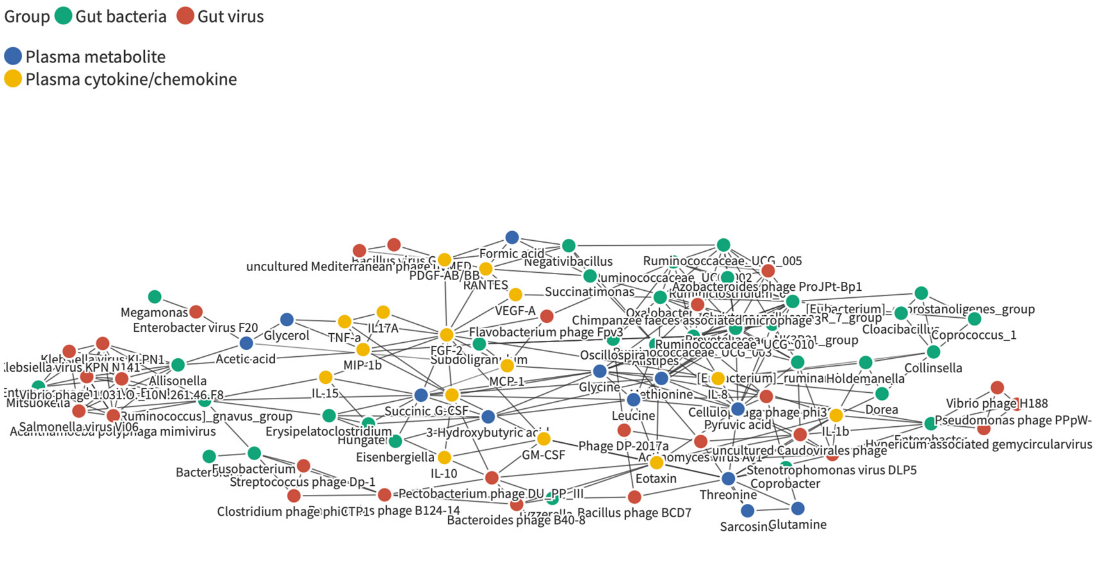

Figure S6. Correlation networks of candidates in all subjects.

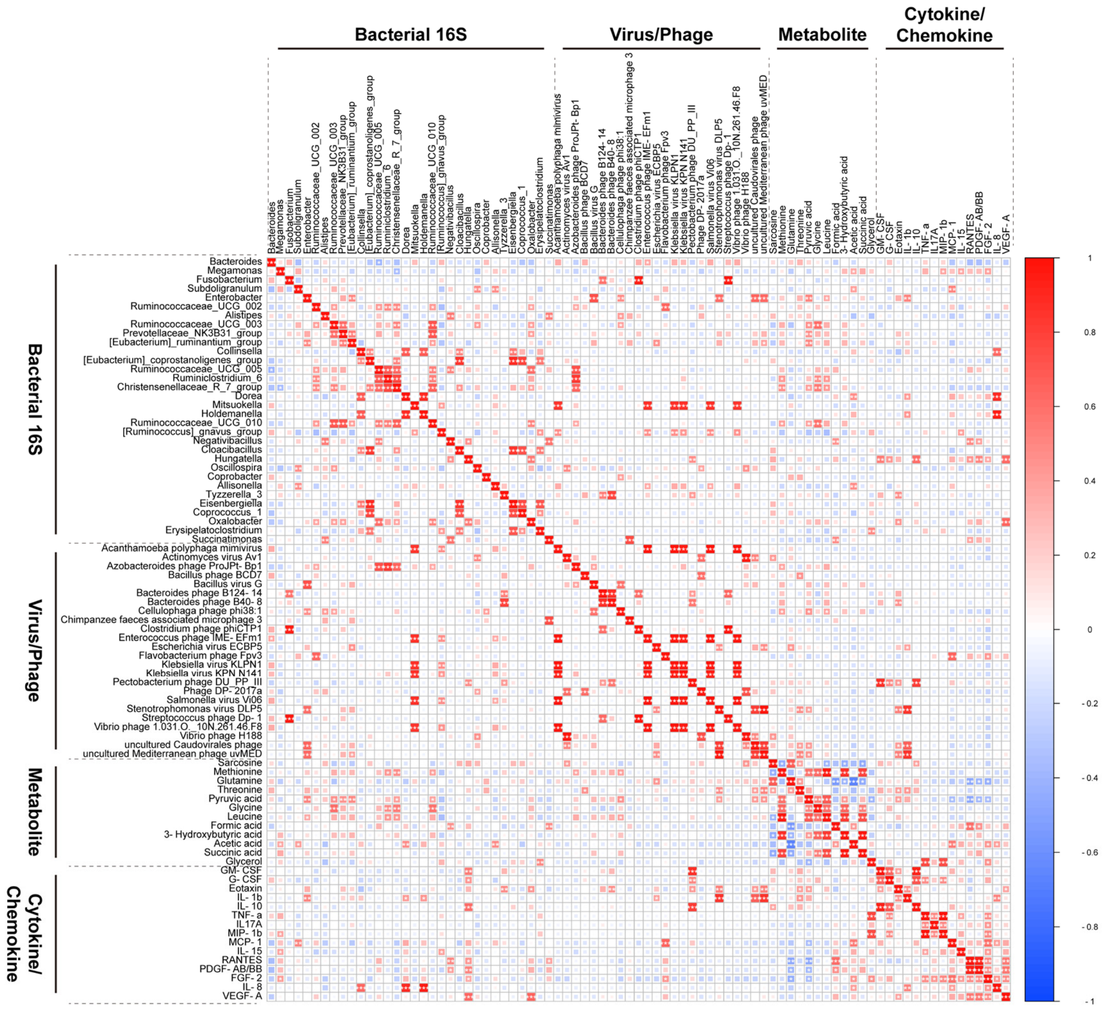

Figure S7. Joint correlation analysis when including the control and LC cohorts. Pearson correlation analysis of candidates from four data sets, including gut bacterial genus, gut viral species, plasma metabolite, and plasma cytokine/chemokine. The asterisk in the square indicates whether the correlation has a significant difference. *, P<0.05; **, P<0.01; ***, P<0.001.

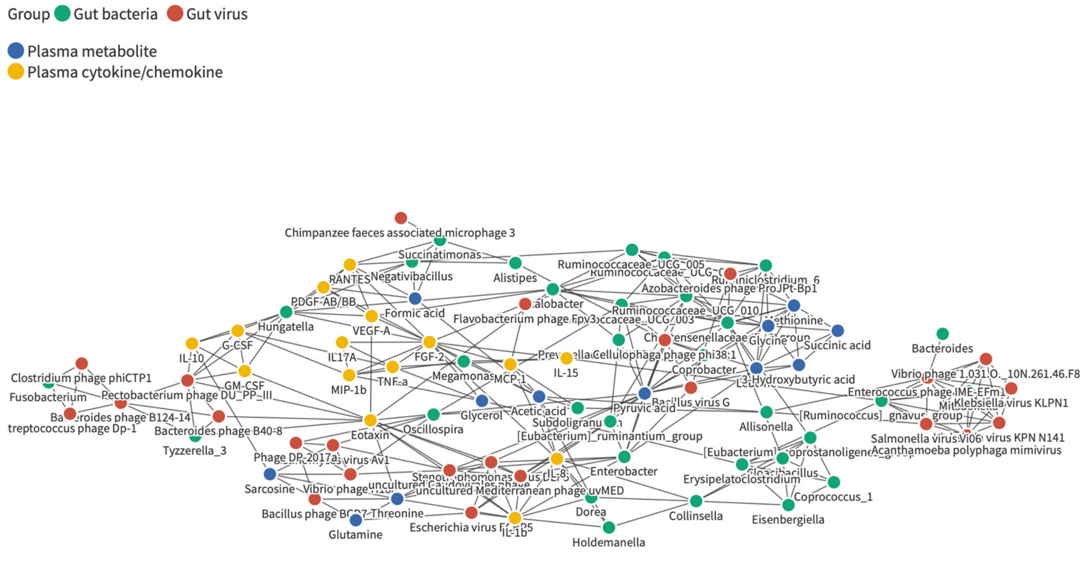

Figure S8. Correlation networks of candidates in the control and LC cohorts.

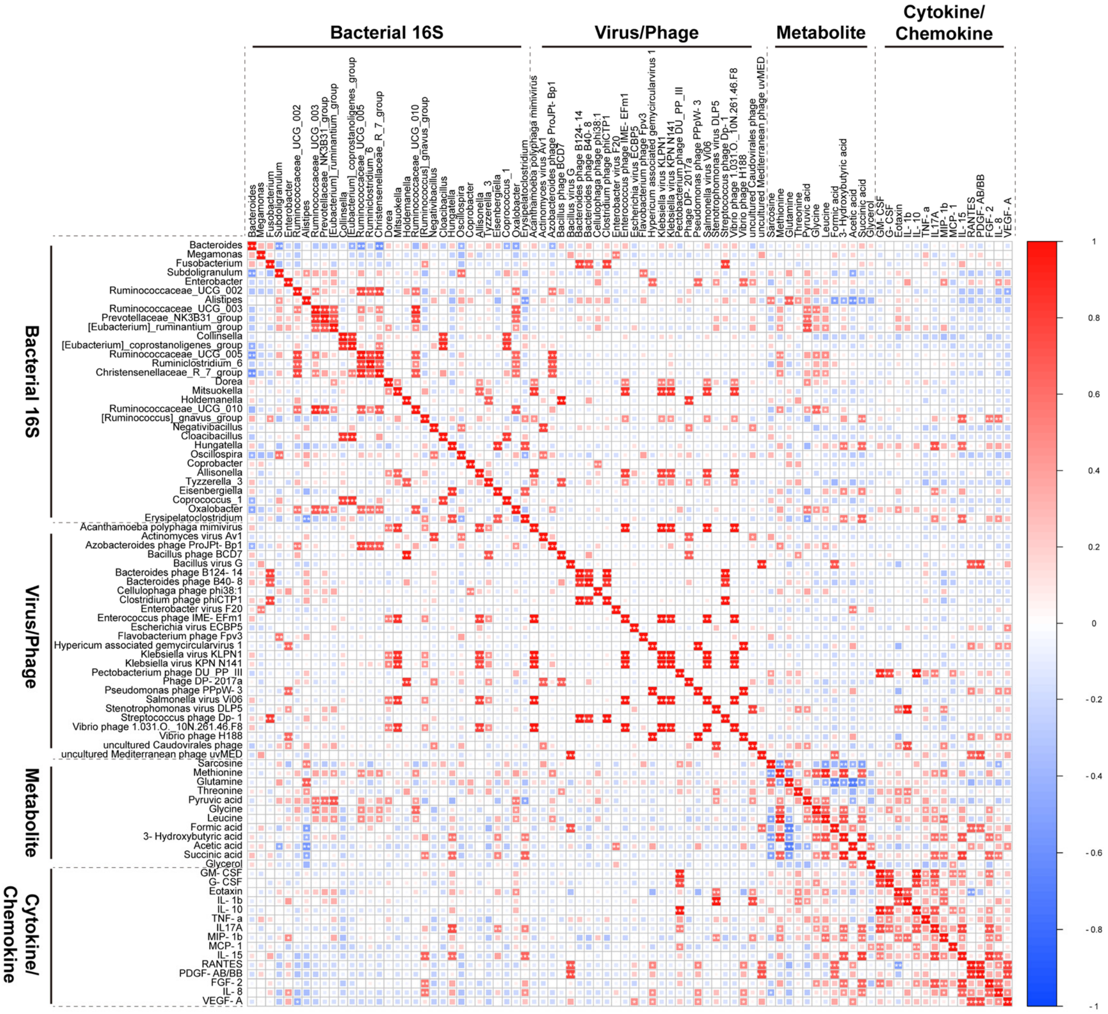

Figure S9. Joint correlation analysis when including the control and HCC cohorts. Pearson correlation analysis of candidates from four data sets, including gut bacterial genus, gut viral species, plasma metabolite, and plasma cytokine/chemokine. The asterisk in the square indicates whether the correlation has a significant difference. *, P<0.05; **, P<0.01; ***, P<0.001.

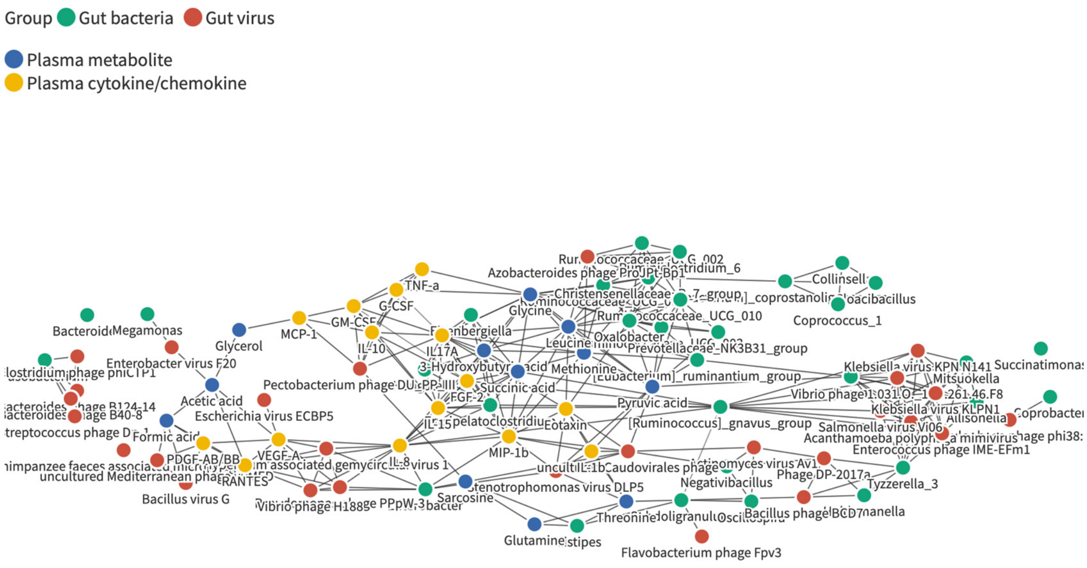

Figure S10. Correlation networks of candidates in the control and HCC cohorts.

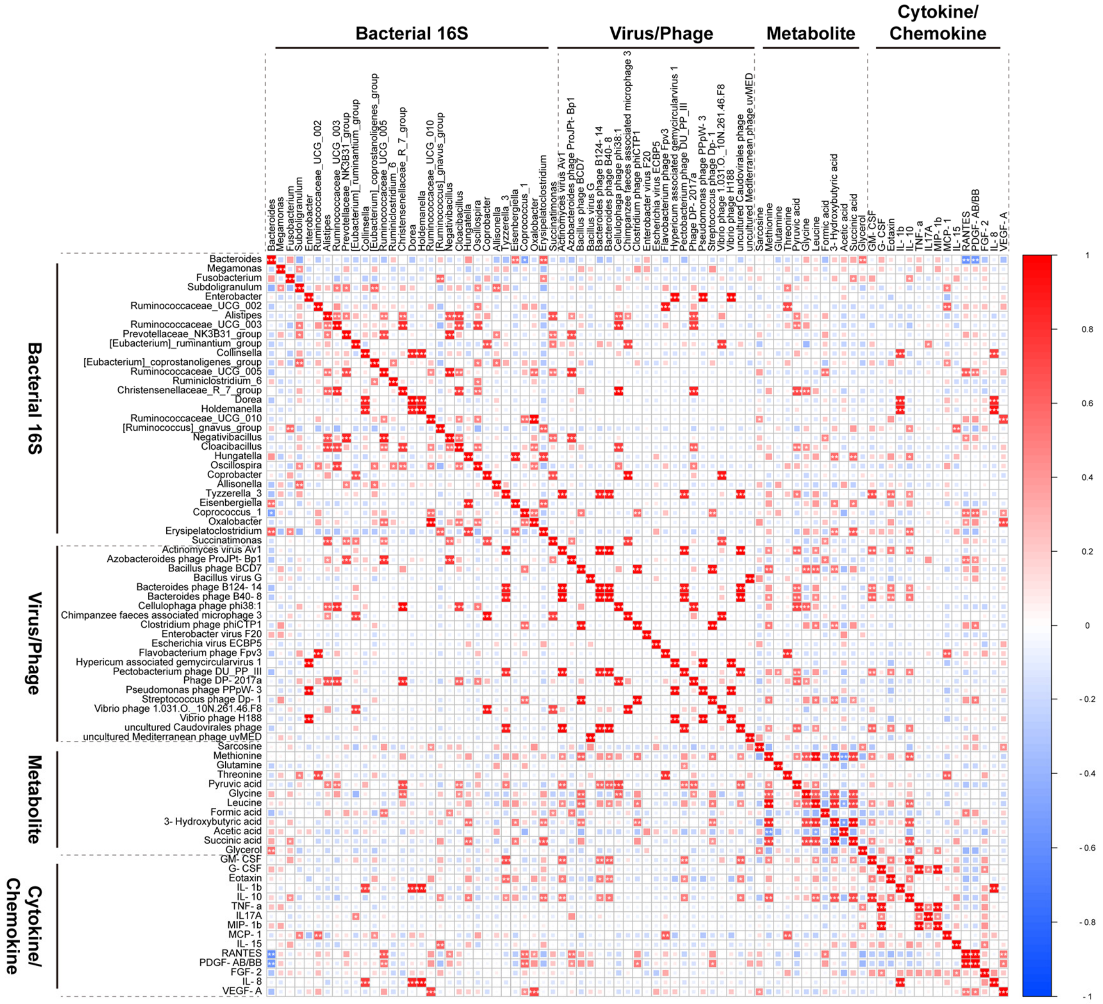

Figure S11. Joint correlation analysis when including the LC and HCC cohorts. Pearson correlation analysis of candidates from four data sets, including gut bacterial genus, gut viral species, plasma metabolite, and plasma cytokine/chemokine. The asterisk in the square indicates whether the correlation has a significant difference. *, P<0.05; **, P<0.01; ***, P<0.001.

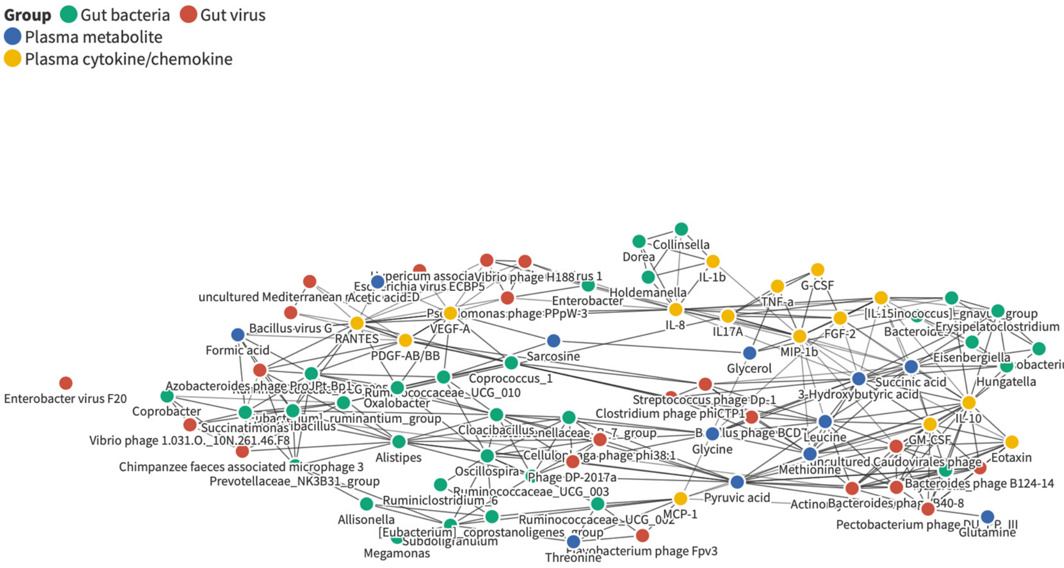

Figure S12. Correlation networks of candidates in the LC and HCC cohorts.

Table S1. Abundance of gut bacterial genus in healthy control, cirrhosis and HCC cohorts.

Taxonomy	Ctrl		LC		HCC		$P_{Ctrl-LC}$	$P_{Ctrl-HCC}$	P_{LC-HCC}
	Mean	SEM	Mean	SEM	Mean	SEM			
Bacteroides	0.2454	0.0467	0.1440	0.0264	0.2917	0.0447	0.086	0.305	**0.012**
Prevotella_9	0.1398	0.0425	0.1775	0.0459	0.0900	0.0530	0.538	0.366	0.151
Faecalibacterium	0.0782	0.0182	0.0972	0.0226	0.0977	0.0179	0.608	0.365	0.658
Phascolarctobacterium	0.0445	0.0086	0.0368	0.0086	0.0516	0.0135	0.485	0.848	0.421
Sutterella	0.0316	0.0074	0.0552	0.0262	0.0406	0.0123	0.761	0.446	0.628
Megamonas	0.0172	0.0060	0.0392	0.0105	0.0175	0.0118	0.067	0.442	**0.018**
Lachnospira	0.0203	0.0043	0.0216	0.0046	0.0307	0.0087	0.964	0.400	0.429
Parabacteroides	0.0149	0.0029	0.0121	0.0039	0.0246	0.0108	0.308	0.723	0.212
Akkermansia	0.0033	0.0019	0.0362	0.0219	0.0045	0.0039	0.268	0.423	0.855
[Eubacterium]_eligens_group	0.0119	0.0038	0.0079	0.0028	0.0250	0.0077	0.624	0.342	0.171
Megasphaera	0.0058	0.0039	0.0306	0.0191	0.0007	0.0005	0.245	0.741	0.485
Lachnoclostridium	0.0150	0.0048	0.0132	0.0023	0.0094	0.0026	0.408	0.529	0.177
Fusobacterium	0.0122	0.0108	0.0063	0.0029	0.0224	0.0102	0.200	**0.020**	0.243
Subdoligranulum	0.0135	0.0028	0.0189	0.0054	0.0003	0.0003	0.750	**0.001**	**<0.001**
Roseburia	0.0139	0.0036	0.0095	0.0028	0.0136	0.0043	0.415	0.938	0.522
Acidaminococcus	0.0014	0.0008	0.0089	0.0044	0.0297	0.0239	0.086	0.417	0.479
Bifidobacterium	0.0167	0.0107	0.0094	0.0046	0.0043	0.0018	0.923	0.612	0.678
Enterobacter	0.0145	0.0099	0.0004	0.0002	0.0193	0.0144	0.437	0.178	**0.044**
Escherichia_Shigella	0.0084	0.0035	0.0124	0.0075	0.0099	0.0038	0.999	0.957	0.957
Ruminococcaceae_UCG_002	0.0148	0.0038	0.0100	0.0048	0.0033	0.0019	0.150	**0.005**	0.138
Alistipes	0.0142	0.0020	0.0108	0.0042	0.0024	0.0010	**0.049**	**0.001**	0.099
Ruminococcaceae_UCG_014	0.0219	0.0194	0.0011	0.0008	0.0026	0.0019	0.121	0.317	0.708

Alloprevotella	0.0071	0.0068	0.0187	0.0162	0.0000	0.0000	0.892	0.315	0.266
Agathobacter	0.0049	0.0021	0.0161	0.0055	0.0063	0.0032	0.059	0.817	0.059
Prevotella_2	0.0168	0.0094	0.0074	0.0049	0.0000	0.0000	0.701	0.062	0.132
Klebsiella	0.0058	0.0031	0.0169	0.0099	0.0032	0.0026	0.493	0.438	0.870
Olsenella	0.0000	0.0000	0.0000	0.0000	0.0275	0.0274	0.971	0.494	0.520
Paraprevotella	0.0097	0.0036	0.0056	0.0017	0.0022	0.0021	0.813	0.095	0.149
Veillonella	0.0024	0.0017	0.0029	0.0012	0.0178	0.0126	0.161	0.056	0.513
Blautia	0.0039	0.0011	0.0084	0.0032	0.0065	0.0021	0.092	0.094	0.867
Dialister	0.0060	0.0020	0.0070	0.0043	0.0035	0.0034	0.880	0.215	0.274
Ruminococcaceae_UCG_003	0.0072	0.0025	0.0067	0.0021	0.0021	0.0009	0.922	**0.035**	**0.030**
Prevotellaceae_NK3B31_group	0.0109	0.0054	0.0029	0.0021	0.0000	0.0000	0.355	**0.047**	0.253
[Eubacterium]_ruminantium_group	0.0081	0.0028	0.0034	0.0019	0.0003	0.0003	0.132	**0.026**	0.384
Streptococcus	0.0022	0.0014	0.0027	0.0012	0.0101	0.0079	0.247	0.400	0.851
Flavonifractor	0.0025	0.0009	0.0046	0.0017	0.0058	0.0018	0.509	0.054	0.186
Butyricicoccus	0.0030	0.0006	0.0043	0.0012	0.0047	0.0014	0.586	0.418	0.749
Collinsella	0.0036	0.0014	0.0059	0.0018	0.0013	0.0005	0.123	0.196	**0.008**
[Eubacterium]_coprostanoligenes_group	0.0080	0.0044	0.0014	0.0004	0.0007	0.0005	0.490	**0.016**	0.078
Lactobacillus	0.0001	0.0001	0.0067	0.0066	0.0055	0.0037	0.725	0.328	0.512
Lachnospiraceae_UCG_004	0.0029	0.0007	0.0036	0.0005	0.0052	0.0021	0.286	0.950	0.380
Ruminococcus_2	0.0016	0.0006	0.0071	0.0065	0.0018	0.0013	0.750	0.361	0.534
Bilophila	0.0027	0.0006	0.0040	0.0011	0.0027	0.0010	0.596	0.619	0.338
[Eubacterium]_hallii_group	0.0062	0.0055	0.0017	0.0012	0.0002	0.0001	0.278	0.503	0.106
Oscillibacter	0.0032	0.0008	0.0030	0.0007	0.0017	0.0010	0.602	0.052	0.097
Fusicatenibacter	0.0015	0.0005	0.0034	0.0012	0.0038	0.0020	0.309	0.712	0.596
Ruminococcaceae_UCG_005	0.0041	0.0017	0.0027	0.0015	0.0002	0.0001	0.092	**0.004**	0.163

Ruminococcaceae_NK4A214_group	0.0029	0.0011	0.0026	0.0011	0.0020	0.0020	0.146	0.068	0.295
Ruminiclostridium_6	0.0043	0.0035	0.0003	0.0003	0.0028	0.0028	**0.015**	**0.025**	0.939
Parasutterella	0.0022	0.0008	0.0027	0.0022	0.0016	0.0011	0.234	0.239	0.909
Christensenellaceae_R_7_group	0.0044	0.0018	0.0009	0.0007	0.0000	0.0000	0.232	**0.008**	0.119
Ruminiclostridium_9	0.0012	0.0005	0.0020	0.0008	0.0034	0.0014	0.850	0.402	0.321
Haemophilus	0.0009	0.0005	0.0026	0.0020	0.0026	0.0014	0.405	0.234	0.659
Anaerostipes	0.0013	0.0010	0.0036	0.0021	0.0005	0.0002	0.183	0.765	0.380
Dorea	0.0008	0.0002	0.0036	0.0016	0.0003	0.0001	0.069	0.119	**0.002**
Lachnospiraceae_UCG_003	0.0012	0.0012	0.0002	0.0002	0.0044	0.0031	0.984	0.246	0.244
Lachnospiraceae_UCG_010	0.0020	0.0005	0.0011	0.0004	0.0018	0.0007	0.181	0.329	0.830
Desulfovibrio	0.0017	0.0009	0.0024	0.0016	0.0001	0.0001	0.127	0.068	0.648
Romboutsia	0.0005	0.0002	0.0037	0.0025	0.0001	0.0000	0.949	0.162	0.151
Ruminococcus_1	0.0015	0.0005	0.0013	0.0007	0.0018	0.0012	0.212	0.222	0.916
Erysipelotrichaceae_UCG_003	0.0015	0.0005	0.0018	0.0010	0.0008	0.0004	0.780	0.281	0.413
Butyricimonas	0.0014	0.0005	0.0016	0.0009	0.0010	0.0006	0.084	0.122	0.994
Tyzzerella	0.0007	0.0005	0.0005	0.0004	0.0037	0.0024	0.550	**0.003**	**0.018**
Mitsuokella	0.0029	0.0019	0.0000	0.0000	0.0000	0.0000	**0.048**	0.078	1.000
Odoribacter	0.0014	0.0004	0.0010	0.0004	0.0010	0.0005	0.445	0.087	0.308
Holdemanella	0.0001	0.0001	0.0029	0.0024	0.0000	0.0000	0.120	0.207	**0.009**
Ruminococcaceae_UCG_010	0.0024	0.0016	0.0000	0.0000	0.0000	0.0000	**0.017**	**0.042**	0.921
Barnesiella	0.0011	0.0005	0.0010	0.0005	0.0006	0.0006	0.615	0.115	0.265
Prevotellaceae_UCG_003	0.0022	0.0015	0.0000	0.0000	0.0000	0.0000	0.111	0.155	1.000
Ruminococcaceae_UCG_013	0.0005	0.0001	0.0004	0.0002	0.0019	0.0011	0.379	0.809	0.311
UBA1819	0.0011	0.0005	0.0005	0.0002	0.0005	0.0002	0.084	0.845	0.184
Prevotellaceae_Ga6A1_group	0.0006	0.0006	0.0014	0.0014	0.0000	0.0000	0.926	0.488	0.443

Ruminiclostridium_5	0.0010	0.0004	0.0004	0.0001	0.0004	0.0001	0.209	0.716	0.455
Coprococcus_2	0.0001	0.0001	0.0009	0.0006	0.0008	0.0008	0.513	0.507	0.219
[Ruminococcus]_gnavus_group	0.0003	0.0002	0.0006	0.0002	0.0007	0.0004	0.462	**0.027**	0.126
Coprococcus_3	0.0004	0.0001	0.0006	0.0003	0.0001	0.0001	0.925	0.104	0.091
[Ruminococcus]_torques_group	0.0005	0.0002	0.0005	0.0002	0.0002	0.0001	0.380	0.444	0.126
Negativibacillus	0.0002	0.0001	0.0008	0.0006	0.0001	0.0001	0.576	0.133	**0.048**
Ruminococcaceae_UCG_004	0.0006	0.0003	0.0003	0.0001	0.0004	0.0002	0.812	0.581	0.737
Cloacibacillus	0.0007	0.0005	0.0003	0.0002	0.0000	0.0000	0.284	**0.041**	0.284
[Eubacterium]_ventriosum_group	0.0007	0.0002	0.0002	0.0001	0.0002	0.0001	0.594	0.372	0.680
Tyzzerella_4	0.0001	0.0001	0.0005	0.0004	0.0007	0.0003	0.771	0.115	0.070
Lachnospiraceae_ND3007_group	0.0003	0.0001	0.0004	0.0001	0.0005	0.0002	0.269	0.530	0.724
Holdemania	0.0003	0.0000	0.0006	0.0003	0.0002	0.0001	0.811	0.522	0.673
Hungatella	0.0001	0.0001	0.0001	0.0001	0.0010	0.0004	0.184	**<0.001**	**0.008**
Oscillospira	0.0005	0.0001	0.0003	0.0001	0.0001	0.0001	0.259	**0.028**	0.239
Coprobacter	0.0006	0.0004	0.0001	0.0001	0.0001	0.0001	**0.010**	**0.034**	0.871
Clostridium_sensu_stricto_1	0.0002	0.0001	0.0002	0.0001	0.0006	0.0003	0.849	0.313	0.245
Enterorhabdus	0.0007	0.0007	0.0000	0.0000	0.0000	0.0000	0.990	0.467	0.465
CAG_56	0.0002	0.0001	0.0002	0.0001	0.0006	0.0004	0.628	0.862	0.550
Lachnospiraceae_UCG_001	0.0001	0.0001	0.0001	0.0001	0.0008	0.0007	0.422	0.799	0.338
Allisonella	0.0002	0.0001	0.0005	0.0002	0.0001	0.0001	0.169	0.370	**0.036**
Family_XIII_AD3011_group	0.0002	0.0001	0.0003	0.0003	0.0003	0.0003	0.165	0.078	0.604
Adlercreutzia	0.0005	0.0004	0.0002	0.0001	0.0001	0.0000	0.774	0.202	0.314
Tyzzerella_3	0.0003	0.0001	0.0003	0.0002	0.0000	0.0000	0.354	**0.027**	0.171
Bacteroidales_bacterium_55_9	0.0006	0.0006	0.0000	0.0000	0.0000	0.0000	0.266	0.321	1.000
Eggerthella	0.0002	0.0002	0.0002	0.0001	0.0003	0.0001	0.291	0.108	0.512

Prevotella	0.0000	0.0000	0.0000	0.0000	0.0008	0.0008	0.683	0.201	0.105
Intestinimonas	0.0002	0.0001	0.0002	0.0001	0.0001	0.0001	0.070	0.058	0.650
Catenibacterium	0.0002	0.0001	0.0002	0.0002	0.0001	0.0001	0.492	0.389	0.807
Prevotella_7	0.0000	0.0000	0.0005	0.0005	0.0000	0.0000	0.912	0.703	0.780
Eisenbergiella	0.0001	0.0001	0.0000	0.0000	0.0006	0.0004	0.889	**0.005**	**0.004**
Phocea	0.0002	0.0001	0.0002	0.0001	0.0001	0.0001	0.921	0.611	0.556
[Ruminococcus]_gauvreauii_group	0.0002	0.0001	0.0002	0.0001	0.0001	0.0001	0.976	0.129	0.141
Prevotellaceae_UCG_001	0.0000	0.0000	0.0003	0.0003	0.0003	0.0003	0.160	0.347	0.757
Fournierella	0.0001	0.0001	0.0002	0.0002	0.0001	0.0001	0.625	0.644	0.375
Coprococcus_1	0.0002	0.0001	0.0001	0.0000	0.0000	0.0000	0.100	**0.006**	0.205
Anaerotruncus	0.0002	0.0001	0.0001	0.0000	0.0001	0.0001	0.757	0.791	0.594
CAG_352	0.0003	0.0003	0.0000	0.0000	0.0000	0.0000	0.312	0.454	0.881
Oxalobacter	0.0002	0.0001	0.0001	0.0001	0.0000	0.0000	0.056	**0.014**	0.456
Senegalimassilia	0.0001	0.0000	0.0001	0.0001	0.0001	0.0001	0.222	0.110	0.615
Ruminococcaceae_UCG_009	0.0001	0.0001	0.0001	0.0000	0.0001	0.0001	0.859	0.838	0.720
Erysipelatoclostridium	0.0000	0.0000	0.0001	0.0000	0.0003	0.0001	0.346	**0.009**	0.083
Enterococcus	0.0002	0.0002	0.0000	0.0000	0.0000	0.0000	0.607	0.345	0.632
Citrobacter	0.0002	0.0002	0.0000	0.0000	0.0001	0.0001	0.414	0.703	0.273
Peptococcus	0.0001	0.0001	0.0001	0.0000	0.0001	0.0000	0.910	0.707	0.786
Victivallis	0.0002	0.0001	0.0001	0.0000	0.0000	0.0000	0.734	0.108	0.197
Succinatimonas	0.0000	0.0000	0.0002	0.0001	0.0000	0.0000	**0.035**	1.000	0.063
Clostridium_sp_K4410MGS_306	0.0002	0.0002	0.0000	0.0000	0.0000	0.0000	0.266	0.321	1.000
Howardella	0.0001	0.0001	0.0000	0.0000	0.0001	0.0001	0.409	0.403	0.921
Lactococcus	0.0001	0.0001	0.0000	0.0000	0.0001	0.0001	0.645	0.309	0.549
[Eubacterium]_xylanophilum_group	0.0001	0.0001	0.0000	0.0000	0.0001	0.0001	0.181	0.374	0.765

Anaeroglobus	0.0000	0.0000	0.0000	0.0000	0.0003	0.0003	1.000	0.112	0.117
Helicobacter	0.0000	0.0000	0.0000	0.0000	0.0003	0.0003	1.000	0.112	0.117
Selenomonas	0.0002	0.0002	0.0000	0.0000	0.0000	0.0000	0.266	0.321	1.000
Pseudoflavonifractor	0.0000	0.0000	0.0000	0.0000	0.0002	0.0002	0.199	0.873	0.330
Mailhella	0.0000	0.0000	0.0002	0.0001	0.0000	0.0000	0.150	0.371	0.700
Methanobrevibacter	0.0002	0.0002	0.0000	0.0000	0.0000	0.0000	0.266	0.321	1.000
Pyramidobacter	0.0001	0.0000	0.0000	0.0000	0.0001	0.0001	0.383	0.392	0.938
Intestinibacter	0.0000	0.0000	0.0000	0.0000	0.0001	0.0001	0.491	0.259	0.611
Rikenellaceae_RC9_gut_group	0.0001	0.0001	0.0000	0.0000	0.0000	0.0000	0.266	0.321	1.000
Actinomyces	0.0001	0.0001	0.0000	0.0000	0.0000	0.0000	0.581	0.771	0.843
Comamonas	0.0001	0.0001	0.0000	0.0000	0.0000	0.0000	0.188	0.814	0.353
Weissella	0.0001	0.0001	0.0001	0.0000	0.0000	0.0000	0.923	0.195	0.173
Sellimonas	0.0000	0.0000	0.0000	0.0000	0.0001	0.0001	0.959	0.093	0.088

Ctrl, control group; LC, liver cirrhosis group; HCC, hepatocellular carcinoma group. P values were obtained using Kruskal-Wallis test, and <0.05 was considered significant. Those with statistical significance were labeled with bold.

Table S2. Abundance of gut virus species in the healthy control, cirrhosis and HCC cohorts.

Taxonomy	Ctrl		LC		HCC		$P_{Ctrl-LC}$	$P_{Ctrl-HCC}$	P_{LC-HCC}
	Mean	SEM	Mean	SEM	Mean	SEM			
Acanthamoeba polyphaga mimivirus	0.0001	0.0001	0.0000	0.0000	0.0000	0.0000	**0.021**	**0.039**	1.000
Acinetobacter phage Acj9	0.0000	0.0000	0.0034	0.0034	0.0000	0.0000	0.938	0.759	0.815
Acinetobacter virus LZ35	0.0000	0.0000	0.0002	0.0002	0.0000	0.0000	0.926	0.488	0.443
Actinomyces virus Av1	0.0012	0.0012	0.0000	0.0000	0.0000	0.0000	0.164	**0.039**	0.417
Aeromonas phage phiA8-29	0.0006	0.0006	0.0002	0.0002	0.0000	0.0000	0.539	0.240	0.536
Arthrobacter phage Niktson	0.0002	0.0002	0.0000	0.0000	0.0000	0.0000	0.317	0.077	0.387
Arthrobacter virus Captnmurica	0.0000	0.0000	0.0009	0.0009	0.0000	0.0000	0.649	0.493	0.782
Azobacteroides phage ProJPt-Bp1	0.0020	0.0009	0.0004	0.0002	0.0001	0.0001	0.102	**0.024**	0.433
Bacillus phage BCD7	0.0193	0.0193	0.0000	0.0000	0.0000	0.0000	0.064	**0.036**	0.658
Bacillus phage PBC2	0.0001	0.0001	0.0000	0.0000	0.0003	0.0003	0.374	0.731	0.658
Bacillus phage SP-10	0.0008	0.0007	0.0006	0.0005	0.0001	0.0001	0.223	0.242	0.935
Bacillus phage VMY22	0.0000	0.0000	0.0381	0.0380	0.0000	0.0000	0.847	0.327	0.255
Bacillus virus B103	0.0000	0.0000	0.0006	0.0005	0.0000	0.0000	0.890	0.689	0.785
Bacillus virus G	0.0005	0.0003	0.0000	0.0000	0.0037	0.0028	0.270	0.248	**0.035**
Bacillus virus GA1	0.0004	0.0004	0.0000	0.0000	0.0000	0.0000	0.329	0.703	0.629
Bacillus virus Moonbeam	0.0000	0.0000	0.0004	0.0004	0.0000	0.0000	0.812	0.646	0.807
Bacillus virus SP15	0.0003	0.0003	0.0000	0.0000	0.0000	0.0000	0.787	0.430	0.588
Bacillus virus Shbh1	0.0004	0.0004	0.0000	0.0000	0.0000	0.0000	0.472	0.665	0.837
Bacillus virus vB_BsuM-Goe2	0.0001	0.0001	0.0000	0.0000	0.0000	0.0000	0.266	0.321	1.000
Bacteriophage sp.	0.0020	0.0015	0.0002	0.0002	0.0005	0.0003	0.184	0.495	0.621
Bacteroides phage B124-14	0.0008	0.0007	0.0012	0.0009	0.0001	0.0001	0.196	**0.022**	0.262
Bacteroides phage B40-8	0.0009	0.0005	0.0020	0.0018	0.0000	0.0000	0.102	**0.005**	0.175

Cafeteria roenbergensis virus	0.0012	0.0012	0.0000	0.0000	0.0000	0.0000	0.765	0.898	0.892
Cellulophaga phage phi14:2	0.0051	0.0032	0.0300	0.0183	0.0499	0.0493	0.081	0.107	0.956
Cellulophaga phage phi19:1	0.0036	0.0036	0.0000	0.0000	0.0000	0.0000	0.266	0.321	1.000
Cellulophaga phage phi38:1	0.0006	0.0004	0.0003	0.0003	0.0000	0.0000	0.088	**0.004**	0.176
Cellulophaga virus Cba172	0.0001	0.0001	0.0000	0.0000	0.0000	0.0000	0.266	0.321	1.000
Cellulophaga virus ST	0.0001	0.0001	0.0011	0.0011	0.0000	0.0000	0.100	0.093	0.832
Chicken stool-associated gemycircularvirus	0.0000	0.0000	0.0000	0.0000	0.0001	0.0001	0.312	0.886	0.303
Chimpanzee faeces associated microphage 3	0.0000	0.0000	0.0004	0.0003	0.0000	0.0000	**0.035**	1.000	0.063
Citrobacter phage CVT22	0.0000	0.0000	0.0018	0.0012	0.0000	0.0000	0.235	0.959	0.273
Clostridium phage phiCP39-O	0.0000	0.0000	0.0001	0.0001	0.0000	0.0000	0.303	0.132	0.560
Clostridium phage phiCTP1	0.0185	0.0163	0.0001	0.0001	0.0000	0.0000	0.168	**0.023**	0.304
Croceibacter phage P2559S	0.0024	0.0016	0.0069	0.0042	0.0070	0.0068	0.904	0.275	0.331
Croceibacter phage P2559Y	0.0001	0.0001	0.0059	0.0035	0.0847	0.0847	0.759	0.122	0.209
Cyanophage P-RSM1	0.0000	0.0000	0.0002	0.0002	0.0000	0.0000	0.926	0.488	0.443
Deep-sea thermophilic phage D6E	0.0000	0.0000	0.0000	0.0000	0.0005	0.0005	1.000	0.112	0.117
Dickeya virus BF25-12	0.0000	0.0000	0.0000	0.0000	0.0002	0.0002	1.000	0.112	0.117
Eel River basin pequenovirus	0.0001	0.0001	0.0001	0.0000	0.0000	0.0000	0.533	0.652	0.918
Enterobacter phage Arya	0.0000	0.0000	0.0000	0.0000	0.0015	0.0015	0.973	0.208	0.203
Enterobacter virus F20	0.0000	0.0000	0.0000	0.0000	0.0044	0.0044	1.000	**0.023**	**0.025**
Enterococcus phage EF1	0.0083	0.0065	0.0038	0.0038	0.0000	0.0000	0.140	0.175	0.967
Enterococcus phage IME-EFm1	0.0003	0.0003	0.0000	0.0000	0.0000	0.0000	**0.048**	0.078	1.000
Enterococcus phage IME-EFm5	0.0002	0.0002	0.0000	0.0000	0.0000	0.0000	0.939	0.303	0.278
Enterococcus virus EFP01	0.0001	0.0001	0.0010	0.0010	0.0000	0.0000	0.214	0.598	0.106
Escherichia phage K1-ind(3)	0.0000	0.0000	0.0005	0.0005	0.0000	0.0000	0.926	0.488	0.443
Escherichia phage vB_EcoS Sa179lw	0.0092	0.0092	0.0000	0.0000	0.0000	0.0000	0.228	0.113	0.615

Escherichia virus ECBP5	0.0000	0.0000	0.0000	0.0000	0.0093	0.0093	0.178	0.306	**0.028**
Escherichia virus Golestan	0.0003	0.0003	0.0000	0.0000	0.0001	0.0001	0.414	0.703	0.273
Escherichia virus IME08	0.0003	0.0003	0.0000	0.0000	0.0000	0.0000	0.111	0.155	1.000
Escherichia virus K1ind1	0.0001	0.0001	0.0000	0.0000	0.0000	0.0000	0.266	0.321	1.000
Escherichia virus LM33P1	0.0012	0.0012	0.0000	0.0000	0.0000	0.0000	0.266	0.321	1.000
Flavobacterium phage Fpv3	0.0000	0.0000	0.0009	0.0007	0.0000	0.0000	0.843	0.053	**0.022**
Geobacillus virus E3	0.0012	0.0009	0.0000	0.0000	0.0000	0.0000	0.243	0.756	0.471
Gokushovirus MK-2017	0.0001	0.0001	0.0005	0.0005	0.0000	0.0000	0.641	0.841	0.832
Gokushovirus WZ-2015a	0.1806	0.0593	0.2105	0.0819	0.1340	0.0984	0.493	0.065	0.224
Grapevine geminivirus A	0.0000	0.0000	0.0002	0.0002	0.0000	0.0000	0.160	0.347	0.757
Human gokushovirus	0.0010	0.0007	0.0000	0.0000	0.0000	0.0000	0.120	0.265	0.790
Human gut gokushovirus	0.1307	0.0448	0.1817	0.0750	0.1349	0.1019	0.706	0.058	0.065
Human gut microviridae SH-CHD12	0.0889	0.0617	0.1346	0.0738	0.0605	0.0523	0.705	0.064	0.135
Human gut microviridae SH-CHD8	0.0000	0.0000	0.0012	0.0009	0.0000	0.0000	0.466	0.794	0.701
Hypericum associated gemycircularvirus 1	0.0000	0.0000	0.0000	0.0000	0.0007	0.0007	1.000	**0.023**	**0.025**
Klebsiella phage JY917	0.0018	0.0017	0.0000	0.0000	0.0000	0.0000	0.133	0.237	0.877
Klebsiella phage vB_Kpn_F48	0.0000	0.0000	0.0021	0.0021	0.0000	0.0000	0.689	0.168	0.314
Klebsiella virus BO1E	0.0000	0.0000	0.0000	0.0000	0.0047	0.0047	1.000	0.112	0.117
Klebsiella virus F19	0.0000	0.0000	0.0000	0.0000	0.0026	0.0026	1.000	0.112	0.117
Klebsiella virus IME205	0.0003	0.0003	0.0000	0.0000	0.0000	0.0000	0.266	0.321	1.000
Klebsiella virus K11	0.0003	0.0003	0.0000	0.0000	0.0000	0.0000	0.414	0.703	0.273
Klebsiella virus K244	0.0000	0.0000	0.0000	0.0000	0.0066	0.0066	0.407	0.244	0.675
Klebsiella virus KLPN1	0.0019	0.0018	0.0000	0.0000	0.0000	0.0000	**0.048**	0.078	1.000
Klebsiella virus KOX1	0.0000	0.0000	0.0000	0.0000	0.0095	0.0095	0.407	0.244	0.675
Klebsiella virus KPN N141	0.0005	0.0004	0.0000	0.0000	0.0000	0.0000	**0.048**	0.078	1.000

Klebsiella virus KPRio2015	0.0000	0.0000	0.0000	0.0000	0.0027	0.0027	1.000	0.112	0.117
Klebsiella virus Kp1	0.0002	0.0002	0.0000	0.0000	0.0000	0.0000	0.266	0.321	1.000
Klebsiella virus Kp2	0.0000	0.0000	0.0000	0.0000	0.0052	0.0052	0.170	0.324	0.815
Klebsiella virus KpCol1	0.0001	0.0001	0.0000	0.0000	0.0021	0.0021	0.388	0.837	0.335
Klebsiella virus KpV522	0.0021	0.0021	0.0000	0.0000	0.0119	0.0119	0.510	0.196	0.063
Klebsiella virus KpV74	0.0000	0.0000	0.0000	0.0000	0.0060	0.0060	1.000	0.112	0.117
Klebsiella virus KpV763	0.0002	0.0002	0.0000	0.0000	0.0000	0.0000	0.990	0.467	0.465
Klebsiella virus KpV767	0.0003	0.0003	0.0000	0.0000	0.0000	0.0000	0.266	0.321	1.000
Klebsiella virus MezzoGao	0.0019	0.0018	0.0000	0.0000	0.0045	0.0031	0.312	0.886	0.303
Klebsiella virus PKP126	0.0019	0.0019	0.0000	0.0000	0.0103	0.0103	0.437	0.637	0.250
Klebsiella virus PRA33	0.0002	0.0002	0.0000	0.0000	0.0000	0.0000	0.266	0.321	1.000
Klebsiella virus SU503	0.0000	0.0000	0.0000	0.0000	0.0117	0.0117	0.407	0.244	0.675
Klebsiella virus Sushi	0.0022	0.0021	0.0000	0.0000	0.0000	0.0000	0.076	0.413	0.451
Lactobacillus phage P1174	0.0001	0.0001	0.0000	0.0000	0.0000	0.0000	0.266	0.321	1.000
Lactobacillus virus Lb338-1	0.0000	0.0000	0.0001	0.0001	0.0000	0.0000	0.926	0.488	0.443
Lactobacillus virus Semele	0.0003	0.0003	0.0000	0.0000	0.0000	0.0000	0.105	0.980	0.161
Lactococcus phage AM6	0.0000	0.0000	0.0070	0.0070	0.0000	0.0000	0.912	0.703	0.780
Lactococcus virus c2	0.0000	0.0000	0.0001	0.0001	0.0000	0.0000	0.926	0.488	0.443
Methylophilaceae phage P19250A	0.0000	0.0000	0.0010	0.0010	0.0000	0.0000	0.939	0.303	0.278
Moumouvirus	0.0002	0.0002	0.0000	0.0000	0.0009	0.0009	0.199	0.873	0.330
Mycobacterium phage MkaliMitinis3	0.0003	0.0002	0.0000	0.0000	0.0000	0.0000	0.252	0.119	0.597
Ochrobactrum phage POA1180	0.0000	0.0000	0.0004	0.0004	0.0000	0.0000	0.803	0.896	0.728
Ostreococcus lucimarinus virus 2	0.0001	0.0001	0.0000	0.0000	0.0000	0.0000	0.266	0.321	1.000
Parabacteroides phage YZ-2015a	0.0001	0.0000	0.0003	0.0003	0.0001	0.0001	0.544	0.180	0.430
Parabacteroides phage YZ-2015b	0.0000	0.0000	0.0063	0.0062	0.0000	0.0000	0.820	0.906	0.751

Pectinobacterium virus PEAT2	0.0010	0.0010	0.0000	0.0000	0.0001	0.0001	0.414	0.703	0.273
Pectobacterium phage DU_PP_III	0.0678	0.0590	0.0790	0.0575	0.0000	0.0000	0.864	**0.023**	**0.037**
Pectobacterium virus PP99	0.0000	0.0000	0.0000	0.0000	0.0035	0.0035	0.988	0.909	0.900
Phage DP-2017a	0.0255	0.0160	0.0149	0.0090	0.0031	0.0031	0.294	**0.020**	0.172
Poophage MBI-2016a	0.0486	0.0296	0.0016	0.0010	0.0000	0.0000	0.067	0.135	0.891
Pseudomonas phage GP100	0.0000	0.0000	0.0007	0.0007	0.0000	0.0000	0.926	0.488	0.443
Pseudomonas phage PPpW-3	0.0000	0.0000	0.0000	0.0000	0.0001	0.0001	1.000	**0.023**	**0.025**
Pseudomonas virus Bjorn	0.0000	0.0000	0.0007	0.0007	0.0000	0.0000	0.235	1.000	0.296
Pseudomonas virus EL	0.0000	0.0000	0.0002	0.0002	0.0000	0.0000	0.235	1.000	0.296
Pseudomonas virus KIL4	0.0002	0.0001	0.0000	0.0000	0.0000	0.0000	0.252	0.119	0.597
Pseudomonas virus PAKP1	0.0022	0.0021	0.0000	0.0000	0.0000	0.0000	0.365	0.367	0.926
Pseudomonas virus tf	0.0000	0.0000	0.0009	0.0009	0.0000	0.0000	0.926	0.488	0.443
Ralstonia virus RsoP1EGY	0.0002	0.0002	0.0000	0.0000	0.0000	0.0000	0.188	0.814	0.353
Rhodococcus virus Pepy6	0.0000	0.0000	0.0002	0.0002	0.0000	0.0000	0.235	1.000	0.296
Salmonella phage 64795_sal3	0.0024	0.0024	0.0000	0.0000	0.0000	0.0000	0.266	0.321	1.000
Salmonella phage IME207	0.0057	0.0057	0.0000	0.0000	0.0000	0.0000	0.177	0.757	0.377
Salmonella phage LPST10	0.0046	0.0046	0.0000	0.0000	0.0000	0.0000	0.414	0.703	0.273
Salmonella phage Vi II-E1	0.0042	0.0042	0.0000	0.0000	0.0000	0.0000	0.990	0.467	0.465
Salmonella virus NR01	0.0000	0.0000	0.0000	0.0000	0.0002	0.0002	1.000	0.112	0.117
Salmonella virus SSE121	0.0003	0.0003	0.0019	0.0019	0.0000	0.0000	0.455	0.557	0.216
Salmonella virus Vi06	0.0003	0.0002	0.0000	0.0000	0.0000	0.0000	**0.048**	0.078	1.000
Shigella phage Sf11 SMD-2017	0.0015	0.0015	0.0000	0.0000	0.0000	0.0000	0.266	0.321	1.000
Shigella virus AG3	0.0018	0.0018	0.0000	0.0000	0.0000	0.0000	0.414	0.703	0.273
Shigella virus SFN6B	0.0000	0.0000	0.0000	0.0000	0.0024	0.0024	1.000	0.112	0.117
Staphylococcus phage pSco-10	0.0044	0.0044	0.0001	0.0001	0.0000	0.0000	0.985	0.291	0.290

Staphylococcus phage vB_SauM_0414_108	0.0001	0.0001	0.0000	0.0000	0.0000	0.0000	0.496	0.229	0.556
Staphylococcus virus BS1	0.0001	0.0000	0.0021	0.0018	0.0000	0.0000	0.655	0.355	0.191
Staphylococcus virus SA12	0.0023	0.0021	0.0004	0.0004	0.0000	0.0000	0.436	0.181	0.525
Staphylococcus virus Sextaec	0.0000	0.0000	0.0000	0.0000	0.0020	0.0020	1.000	0.112	0.117
Stenotrophomonas virus DLP5	0.0002	0.0002	0.0000	0.0000	0.0000	0.0000	**0.048**	0.078	1.000
Streptococcus phage Dp-1	0.0024	0.0024	0.0000	0.0000	0.0000	0.0000	**0.028**	**0.016**	0.655
Streptococcus phage Str-PAP-1	0.0000	0.0000	0.0003	0.0003	0.0000	0.0000	0.455	0.557	0.216
Streptococcus virus Cp1	0.0000	0.0000	0.0000	0.0000	0.0001	0.0001	0.992	0.690	0.700
Streptomyces phage BRock	0.0001	0.0001	0.0038	0.0038	0.0000	0.0000	0.264	0.052	0.350
TM7 phage DolZOral124_53_65	0.0024	0.0024	0.0001	0.0001	0.0000	0.0000	0.222	0.201	0.852
Tenacibaculum phage pT24	0.0005	0.0004	0.0001	0.0001	0.0074	0.0074	0.970	0.891	0.919
Tokyovirus A1	0.0005	0.0005	0.0000	0.0000	0.0000	0.0000	0.750	0.206	0.333
Uncultured phage WW-nAnB strain 3	0.0002	0.0002	0.0000	0.0000	0.0000	0.0000	0.266	0.321	1.000
Vibrio phage 1.031.O._10N.261.46.F8	0.0004	0.0003	0.0000	0.0000	0.0000	0.0000	0.119	**0.013**	0.278
Vibrio phage 1.081.O._10N.286.52.C2	0.0001	0.0001	0.0000	0.0000	0.0000	0.0000	0.266	0.321	1.000
Vibrio phage 1.122.A._10N.286.46.F8	0.0003	0.0003	0.0000	0.0000	0.0019	0.0019	0.599	0.904	0.731
Vibrio phage 1.148.O._10N.286.54.A10	0.0002	0.0002	0.0000	0.0000	0.0005	0.0005	0.091	0.533	0.382
Vibrio phage 1.184.A._10N.286.49.A5	0.0000	0.0000	0.0012	0.0012	0.0000	0.0000	0.235	1.000	0.296
Vibrio phage H188	0.0000	0.0000	0.0000	0.0000	0.0003	0.0003	0.295	0.191	**0.027**
Vibrio phage Rostov-1	0.0003	0.0003	0.0000	0.0000	0.0000	0.0000	0.086	0.491	0.404
Vibrio phage pYD38-A	0.0043	0.0043	0.0126	0.0126	0.0000	0.0000	0.208	0.468	0.695
Vibrio virus VEN	0.0002	0.0002	0.0000	0.0000	0.0000	0.0000	0.266	0.321	1.000
Virus Rctr197k	0.0002	0.0002	0.0000	0.0000	0.0000	0.0000	0.188	0.814	0.353
Xanthomonas phage XacN1	0.0001	0.0001	0.0000	0.0000	0.0000	0.0000	0.266	0.321	1.000
uncultured Caudovirales phage	0.0003	0.0002	0.0000	0.0000	0.0000	0.0000	**0.047**	**0.040**	0.779

uncultured Mediterranean phage uvMED	0.0008	0.0006	0.0000	0.0000	0.0036	0.0031	**0.024**	0.309	0.326
uncultured crAssphage	0.1542	0.0756	0.1551	0.0667	0.3204	0.1401	0.096	0.424	0.500

Ctrl, control group; LC, liver cirrhosis group; HCC, hepatocellular carcinoma group. P values were obtained using Kruskal-Wallis test, and <0.05 was considered significant. Those with statistical significance were labeled with bold.
